# Source attribution of Campylobacter jejuni shows variable importance of chicken and ruminants reservoirs in non-invasive and invasive French clinical isolates

**DOI:** 10.1038/s41598-019-44454-2

**Published:** 2019-05-30

**Authors:** Elvire Berthenet, Amandine Thépault, Marianne Chemaly, Katell Rivoal, Astrid Ducournau, Alice Buissonnière, Lucie Bénéjat, Emilie Bessède, Francis Mégraud, Samuel K. Sheppard, Philippe Lehours

**Affiliations:** 1 French National Reference Center for Campylobacters & Helicobacters, Bordeaux, France; 2 Unit of Hygiene and Quality of Poultry & Pork Products, Laboratory of Ploufragan-Plouzané-Niort, French Agency for Food Environmental and Occupational Health & Safety (ANSES), Ploufragan, France; 3 Univ. Bordeaux, INSERM, UMR1053 Bordeaux Research in Translational Oncology, BaRITOn, 33076 Bordeaux, France; 4 The Milner Centre for Evolution, Department of Biology and Biochemistry, University of Bath, Claverton Down, Bath, United Kingdom

**Keywords:** Clinical microbiology, Comparative genomics

## Abstract

*Campylobacter jejuni* is the most common cause of bacterial gastroenteritis worldwide. Mainly isolated from stool samples, *C. jejuni* can also become invasive. *C. jejuni* belongs to the commensal microbiota of a number of hosts, and infection by this bacterium can sometimes be traced back to exposure to a specific source. Here we genome sequenced 200 clinical isolates (2010–2016) and analyzed them with 701 isolate genomes from human infection, chicken, ruminants and the environment to examine the relative contribution of different reservoirs to non-invasive and invasive infection in France. Host-segregating genetic markers that can discriminate *C. jejuni* source were used with STRUCTURE software to probabilistically attribute the source of clinical strains. A self-attribution correction step, based upon the accuracy of source apportionment within each potential reservoir, improved attribution accuracy of clinical strains and suggested an important role for ruminant reservoirs in non-invasive infection and a potentially increased contribution of chicken as a source of invasive isolates. Structured sampling of *Campylobacter* in the clinic and from potential reservoirs provided evidence for variation in the contribution of different infection sources over time and an important role for non-poultry reservoirs in France. This provides a basis for ongoing genomic epidemiology surveillance and targeted interventions.

## Introduction

*Campylobacter jejuni* is one of the most common bacterial enteropathogens in both high and low income countries^1,2^. In clinical microbiology laboratories, *C. jejuni* is mainly isolated from stools, but 1–2% of cultured strains are isolated from blood^3,4^. Infection symptoms vary from watery diarrhea to bloody stools, accompanied by fever, abdominal pain, vomiting and dehydration^5,6^. Post-infectious complications can occur, including Guillain-Barré syndrome^7^. Because of it’s clinical importance, determining the source of *C. jejuni* infection is a high priority. However, this is challenging as *C. jejuni* is part of the commensal microbiota of many mammal and bird species and is commonly isolated from poultry^8,9^, ruminants^9,10^, pigs^11,12^, wild birds^13,14^ and companion animals (dogs and cats)^15^, as well as the environment^16,17^.

In the last two decades, characterization of strain variation within populations, using DNA sequence based methods such as Multi-Locus Sequencing Type (MLST)^18^ and whole genome sequencing (WGS)^19^, has improved understanding of *Campylobacter* ecology, epidemiology and evolution. In particular, the degree to which lineages are associated with different hosts, reflecting segregating genetic variation that has resulted from the physical isolation of populations in discrete niches as well as adaptations that promote survival in a given host^20^. For example, sequence types (STs) belonging to the ST-257 and ST-353 clonal complexes are most commonly isolated from chickens while ST-61 or ST-42 complex isolates are associated with ruminants^14,21^. As such these lineages can be described as host specialists^21,22^. An applied advantage of understanding the genomics of lineage-host association is that the origin of isolates from human infection can potentially be determined by comparison to genome sequenced isolates from putative reservoir sources, and quantitative probabilistic models have been developed for source attribution of clinical strains^23^.

Attribution studies using MLST data have successfully identified the relative contribution of different host reservoirs to human infection and contamination from chicken reservoirs was implicated in several countries^24,25^. However, a limitation to these approaches is that some of the most common strains infecting humans are found in multiple hosts. These ecological generalist strains cannot be easily assigned to one source as recent host transitions erode the signal of host association^26,27^. While this remains a challenging, decreasing costs and increasing availability of large WGS datasets^28^ is improving understanding of the genes and genetic elements that promote *C. jejuni* host adaptation^29,30^ and survival^31,32^ in particular niches. These elements represent candidate markers for source attribution studies and recent work analyzing the pan-genome of 4 *C. jejuni* reference strains in 884 genomes identified 15 host-segregating markers that were used for source attribution of specialist and generalist genotypes^33^. This last method allowed a better accuracy of attributions compared with MLST loci and a higher host segregation of isolates even in host generalist clonal complexes^34^.

Implementing these approaches has highlighted the importance of chickens and ruminants as sources of *C. jejuni* strains that infect people in France^34^. The French National Reference Centre receives strains sent by a network of around 200 clinical laboratories spread all over the French territory. Among these strains, most are isolated from stools, but the number of invasive *C. jejuni* strains, isolated from blood, has been steadily increasing since 2014, and in 2017 bacteraemia cases caused by *C. jejuni* exceeded those caused by *Campylobacter fetus* for the first time. The reason for this increase is not known but some clonal complexes have been associated with invasive disease. For example, ST-677 clonal complex isolates that are a common cause of diarrhoeal disease in Finland^35^ were also a common cause of invasive infection^4^. However, this clonal complex is less common in some other surveyed countries, including France^34^, and it is unclear if particular lineages are over represented in invasive disease.

Source attribution studies are helping to describe the previously cryptic transmission networks of sporadic disease caused by *Campylobacter*. However, effective implementation for epidemiological monitoring is hampered by limitations in attribution study design. First, these studies typically represent epidemiological snapshots representing a discrete period of time, meaning that long term variation and fine scale trends in source-sink dynamics are overlooked. Second, incomplete segregation of genomic markers by source (host) can lead to a weak signal of self-attribution and bias in the overall attribution results. Third, attribution studies typically treat all *Campylobacter* strains equally and, therefore, do not identify the potential sources of strains causing severe infection (i.e. invasive disease). In this study we aimed to improve understanding of the source dynamics of *C. jejuni* infection in France over the last 10 years. Sampling and sequencing (WGS) contemporary isolates (non-invasive and invasive) and analyzing them with available clinical and potential source isolates, we use host-segregating markers^33^ and an enhanced source attribution model incorporating a self-attribution correction step. Our analysis describes fluctuations in contribution of host reservoirs over time. Chicken remained a major contributor to non-invasive and invasive disease but there is evidence of ruminant reservoirs as a source of strains associated with invasive disease.

## Results

### Genetic structure and organization of the dataset

**Figure 1:**
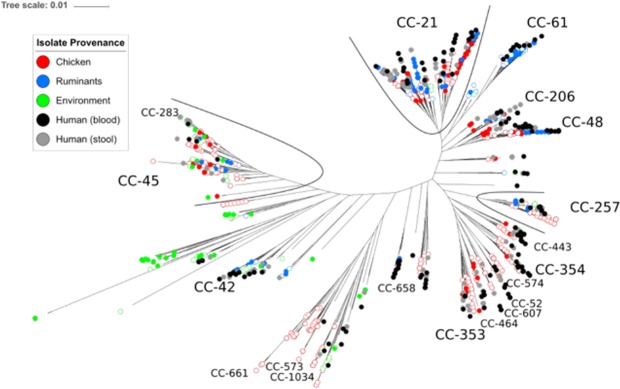
Maximum-likelihood tree based on 1422 concatenated core genes from the 899 strains of *C. jejuni*. Filled circles represent French strains, white circles represent strains from the rest of the world. Clonal complexes obtained from MLST that contain more than 10 strains are labelled in small font. Clonal complexes containing more than 20 strains are labelled in larger font.

Consistent with previous studies^36^, a core-genome tree, based upon 1422 genes shared by at least 90% of sources strains and clinical strains (Fig. 1) showed no evidence of segregation of the French isolates from potential sources compared to those from other countries. Furthermore, clinical strains clustered with strains from potential sources. These two findings confirmed the validity of using a world-wide dataset of source strains to study French clinical isolates. MLST profiles of the complete dataset were concordant with previously published data^22,37–39^, with both host generalist and host specialist clonal complexes. Among the host generalist clonal complexes, CC-21 (176 strains) and CC-45 (92 strains) were the most abundant. Known host specialist clonal complexes were also identified including the chicken-associated CC-353 (35 strains) and CC-354 (31 strains) and ruminant-associated CC-42 (20 strains).

### Self-attribution of isolates from chicken and ruminants reservoirs

**Figure 2:**
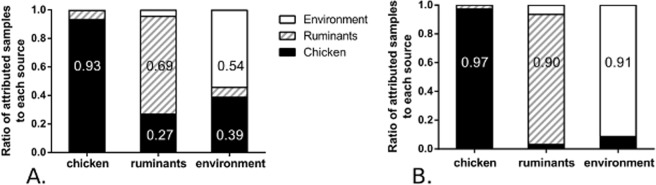
Self-attribution of isolates from chicken, cattle and environment sources. Attribution tests were performed using the STRUCTURE software with 10 replicates. For each sub-dataset of 20 isolates coming from a known source, the proportion of isolates attributed to each reservoir is represented. (**A**) Original self-attribution, uncorrected STRUCTURE results. (**B**) Corrected self-attribution, after correction step based on a system of equations that balances the bias observed in the self-attribution internal results of each attribution test.

The accuracy of the attribution based upon probabilistic assignment of 15 host segregating markers with STRUCTURE was tested using isolates of known origin (self-attribution). A subset of 20 strains from each of the two reservoirs was randomly selected and 10 replicates of attribution tests were performed by comparison to the remaining isolates from the same host. The average probability of each provenance for the subsets of strains was then analyzed (Fig. 2A). The probabilities of correct self-attribution were estimated at 93% for the chicken reservoir, 69% for the ruminant reservoir and 54% for the environment. These results are acceptable for the chicken reservoir, but highlight a bias for the ruminant and environment in favor of the chicken reservoir, with a risk of under-estimation of test isolates attributed to the latter two sources and over-estimation of the proportion of chicken-attributed isolates. Correction of the bias, using the new method based on a system of 3 equations, gave probabilities of correct self-attribution estimated at 97% for the chicken reservoir, 90% for the ruminant reservoir and 91% for the environment reservoir (Fig. 2B). With correct self-attribution of more than 90% for each of the reservoirs, the correction method provides a useful method for attribution of French clinical isolates.

### Attribution of french clinical isolates

**Figure 3:**
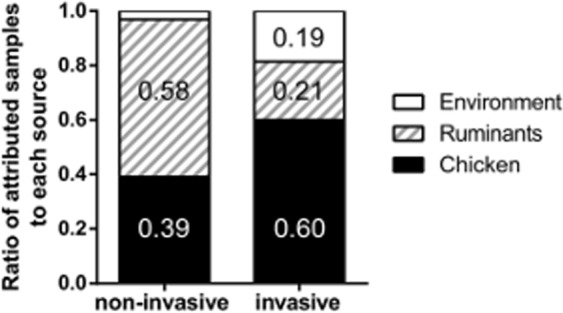
Attribution of French clinical non-invasive (n = 104) and invasive (n = 105) isolates, collected between 2014 and 2016, to isolates from chicken, ruminants and environment sources (352, 136 and 95 isolates respectively). Attribution tests were performed using the STRUCTURE software with 10 replicates. The corrected proportion of isolates attributed to each reservoir is represented after the model correction step based on the observed self-attribution results for each attribution test.

Attribution of the most recent French clinical isolates (from 2014 to 2016) was performed using the 15 host-segregating markers and STRUCTURE software. Corrected attribution results (Fig. 3) show that the proportion of non-invasive isolates attributed to the chicken reservoir (39%) was lower than in previous studies^34^ were 63% of clinical isolates from 2015 were attributed to chicken using the same 15 host-segregating markers. Isolates attributed to ruminants (58%) were predominant in the non-invasive isolates from 2014 to 2016. The proportion of chicken attributed isolates in invasive strains was much higher (60%) compared to non-invasive isolates. The remaining invasive isolates were attributed equally to the ruminants and the environment (21% and 19%, respectively).

**Figure 4:**
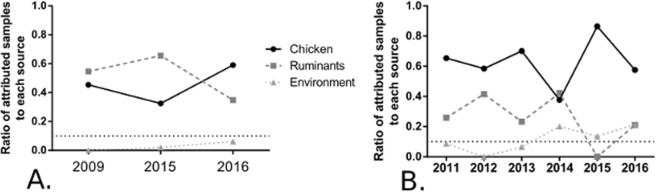
Attribution of French clinical isolates collected between 2009 and 2016 over time based on a collection. Attribution tests were performed using the STRUCTURE software with 10 replicates. A corrected proportion of isolates attributed to each reservoir (after model correction) is represented according to the year of isolation. (**A**) Attribution of French non-invasive clinical isolates collected in 2009 (n = 39), 2015 (n = 78) and 2016 (n = 26). (**B**) Attribution of French invasive clinical isolates collected in 2011 (n = 17), 2012 (n = 18), 2013 (n = 33), 2014 (n = 35), 2015 (n = 33) and 2016 (n = 37).

Attribution of non-invasive strains was performed independently for strains isolated in 2009, 2015 and 2016 (39, 78 and 26 strains, respectively), since a variation in source attribution according to the year of isolation was observed^34^. Corrected attribution results (Fig. 4A) show that the proportion of environment attributed strains remained low (below 10%) over time. The majority of isolates were attributed to ruminants in 2009 and 2015 (54.6% and 64.5%, respectively), but switched to a majority of chicken attributed isolates in 2016 (59%). Attribution of invasive strains was performed independently year-by-year for strains isolated in 2011, 2012, 2013, 2014, 2015 and 2016 (17, 18, 33, 35, 33 and 37 strains respectively). Corrected attribution results (Fig. 4B) show that the proportion of environment attributed strains, increased after 2014 from below 10% (8.8%, 0% and 6.5% for 2011, 2012 and 2013 respectively) to over 10% (20.1%, 13.5% and 21.4% for 2014, 2015 and 2016 respectively). The majority of isolates (57.6% to 86.5%) were attributed to chicken every year except 2014 were only 37.7% of isolates were attributed to chicken.

## Discussion

The continued importance of campylobacteriosis as a major preventable cause of gastroenteritis means that effective monitoring is a high priority in many countries. *C. jejuni* infection is typically associated with contaminated food or drink but as peoples dietary habits vary by country, exposure and source of infection may also vary. This means that source attribution studies in, for example, the UK may not be an accurate reflection of the source of infection elsewhere. In this study we aimed to provide an improved understanding of *C. jejuni* source dynamics in France that incorporated the strengths of: (i) national scale reference laboratory surveillance; (ii) selection of genome-wide host segregating markers; (iii) probabilistic model (STRUCTURE) correction based upon self-attribution. This genomic epidemiology surveillance program was implemented to investigate the French clinical isolates from both invasive and non-invasive infection over the last 10 years.

As in previous studies in France^34^ and other countries^8,23–25,40,41^, chicken is a major source of infection in this study. However, among non-invasive strains isolated between 2014 and 2016, the overall proportion attributed to chicken was lower than previously described, only 39%. Attribution of non-invasive strains to chicken in 2016 increased and even if this trend does not continue, there is ample justification for the continuation of prevention efforts currently in place to control the rates of infection at the slaughterhouse level of the production chain^42^. Efforts to reduce the contamination of chicken at every step of the production chain would also help reducing the risks of campylobacteriosis^42^.

Contaminated chicken is not the only source of campylobacteriosis and previous studies have highlighted ruminants as an important source^8,34,35^. Consistent with this, our study revealed ruminants to be a potentially important source. This could be related to French dietary habits in terms of meat consumption, with a more diverse diet compared to countries such as the United Kingdom^43^. Evidence of the importance of the ruminant reservoir could also be driven by the correction of model bias that was included in the analysis. Indeed, results for attribution in our study showed increased ruminant proportion after the correction step compared with the ruminant proportion before correction (Supp. Fig. 1). Recent chicken-ruminant host transitions leading to incorrect attribution of ruminant strains to chicken could reduce the signal of ruminant attribution in studies that are not correcting the attribution results.

The environment is not thought to be a major reservoir for infection, not least because *C. jejuni* is not thought to thrive outside the host gut. This is a composite niche reflecting contamination from multiple hosts. For this reason, attribution to this source is potentially more complex. However, after model correction self-attribution of environmental strains provided sufficient discrimination for attribution of clinical strains. Before 2014, the proportion of clinical strains attributed to the environment was below 10%, and increased to approximately 20% in 2014 and beyond. This recent increase may suggest that environmental strains reflect another infection reservoir. For example, *C. jejuni* has been isolated in seafood such as mussels in small proportions^17^.

There is evidence for increased incidence of invasive disease caused by *C. jejuni*. This may be related to host factors or mobile genetic elements that confer virulence on particular strains. Neither of these were addressed here, however, it is also possible that certain reservoir sources have increased relative importance as a source of strains associated with invasive disease. In the clinical invasive strains isolated between 2014 and 2016, chicken were the major contributor (60% of cases), with 21% of strains attributed to ruminants and 19% to environment. This varied over time, with an increase in environmental attributed strains after 2014 from about 10% to about 20%, and a drop in the ruminant proportion particularly important in 2015.

Probabilistic attribution models, such the STRUCTURE based method used here, have considerable potential for improving understanding the epidemiology and spread of *Campylobacter* and can form an important part of reference surveillance and targeted interventions. However, there are limitations. First, source reservoirs are identified *a priori* excluding the possibility of attribution to unknown reservoirs. Second, models typically assume that the isolates from a given source are representative. Third, transition of strains between hosts can make definitive attribution difficult. Despite the strength of our method, highlighted by the high rates of self-attribution, we did not reach 100% self-attribution. This means that there is still a risk of erroneous attribution. Finally, source populations of different sizes may effect the probability of attribution of clinical isolates. In this study we used all the available data to maximize the reservoir strains available. As more isolates are sampled and genome sequenced from multiple sources the impact of these limitations will be reduced. Differences observed in attribution results between our study and previous ones could also be a consequence of the evolution of training datasets available to perform attribution studies at different times. Moreover, despite the homogeneous distribution of our source and clinical datasets verified in Fig. 1, there is no concordance of time and space between the source and clinical datasets which could introduce a bias.

In conclusion, this study confirmed not only the importance of the chicken reservoir, but also the importance of ruminants and the environment reservoirs for human campylobacteriosis in France. Furthermore, potential differences in the source of invasive and non-invasive clinical strains suggest that chickens may be a source of more serious infections. This study provides a basis for ongoing genomic epidemiology surveillance of *Campylobacter* in France, and reveal a need for investigation on genomics traits associated with invasive strains that will be carried out using GWAS methods^31,44^.

## Material and Methods

### *C. jejuni* isolates and genome sequencing

The collection of *C. jejuni* genomes from chicken, ruminants and the environment were obtained from previously published studies^33,34^. This collection was comprised of 352, 136 and 95 isolates from chicken, ruminants and the environment, respectively, including isolates from France and from the rest of the world (Supp. Table 1). Consistent with previous studies^23,31,40^, isolates from caecal content, carcass, chicken farms or organs were grouped into a single “chicken” category in order to increase the numbers of strains included as training dataset to increase the efficiency of analyses. Strains from the environment linked to chicken or ruminant farms were included in the chicken or ruminant reservoirs, respectively. Previously published strains of *C. jejuni* isolated from patients in France in 2009 (40 strains)^33,34^ and in 2015 (79 strains)^33^ were included in our dataset. The provenance of the isolates (blood or stools) was traced back and confirmed as stools for 39 of the 40 strains from 2009 and for 78 of the 79 strains from 2015. One isolate from 2015 was isolated from blood. The isolate of unknown provenance from 2009 was not used in our study (Supp. Table 2).

A representative collection of clinical strains comprised 198 clinical strains isolated in France between 2011 and 2016 (Table 1). These isolates were received as single colonies by the CNRCH from French laboratories and hospitals participating in its surveillance network. In this dataset, 63 were sent by private laboratories and 135 were sent by public hospitals spread among 54 of the 102 French departments (Supp. Fig. 2). All invasive strains available to us for the studied years of isolation were selected, and a random selection of non-invasive strains isolated in 2016 was used to complete the collection of non-invasive strains publicly available. Upon reception, stocks of single colonies were maintained at −80 °C in brucella broth with 25% glycerol. Bacterial pellets were digested using MagNA Pure 96 DNA Bacteria Lysis Buffer and proteinase K. DNA extraction was performed on a MagNA Pure 96 System (Roche Applied Science, Manheim, Germany) using the MagNA Pure 96 DNA and Viral NA SV Kit (Roche Applied Science). Quantification and purity checks (260/280 and 260/230 ratios) were determined by spectrophotometry (NanoDrop Technologies, Wilmington, DE, USA) before sequencing (performed by Helixio, Clermont-Ferrand, France). Qubit quantification was carried out prior to sequencing. Library preparation was made using the Nextera XT DNA Library Preparation Kit (Illumina Inc, San Diego, CA, USA) from 1 ng of DNA, and validation of the libraries was performed on the bioanalyzer with the High Sensitivity DNA Assay kit (Agilent, Santa Clara, CA, USA) in order to obtain sizes ranging from 250 to 1,500 bp. Paired-end sequencing was then performed on a NextSeq. 500 (Illumina Inc). Quality was controlled using FastQC v0.11.3^45^. De novo assemblies were produced using SPAdes (v3.10.1)^46^. An average of 20.6 contigs were obtained for the 198 sequenced strains, with a median value of 19 contigs. The average total size was 1,676,574 bp. (Supp. Table 3).

**Table 1:** Clinical *Campylobacter jejuni* isolates from French patients.

Year	Invasive *C. jejuni*	Non-invasive *C. jejuni*
2009	—	39^a^
2011	17^b^	—
2012	18^b^	—
2013	33^b^	—
2014	35^b^	—
2015	1^a^ + 32^b^	78^a^
2016	37^b^	26^b^
Total	**173**	**143**

^a^Previously published isolates.

^b^Newly sequenced isolates.

All of the genomic sequences, and associated information, were stored on a web-based Bacterial Isolates Genomic Sequences database (BIGSdb, http://zoo-dalmore.zoo.ox.ac.uk/)^47^.

### Genetic composition of the dataset

The BLAST algorithm implemented in BIGSdb was used to perform gene-by-gene alignment on the 899 *C. jejuni* genomes of our dataset using the 1,572 coding sequences from the reference strain NCTC 11168 (acc. Number: NC_002163.1). The concatenated alignment obtained for the core genes (present in at least 90% of the strains) was used to produce a phylogenetic tree using FastTree2 software annotated by iTOL v3^48^. MLST typing was performed automatically on all of the 899 strains using a MLST scheme implemented in BIGSdb.

### Preparation of sequences for source attribution

Sequences for the 15 host-segregating markers were downloaded from BIGSdb and used for attribution (Supp. Table 4). The list of 15 host-segregating markers was used to perform a nucleotide BLAST on the 899 strains of our dataset using the genome comparator tool implemented in BIGSdb. The genome comparator tool attributed a unique identification number to each allele of the 15 host-segregating markers. The resulting matrix, identifying the allele present in each strain for each of the 15 host-segregating markers, was then re-formatted and used as input in STRUCTURE^49^.

### Self-attribution of isolates from the 3 putative sources and attribution of french clinical isolates using 15 host-segregating markers

Self-attribution tests were performed using only the 583 training dataset strains from the 3 putative sources. For these self-attribution tests, 20 isolates from each source or reservoir (chicken, ruminants and the environment) were randomly selected to constitute 3 test datasets. Strains belonging to the test datasets were flagged with a 0 using POPFLAG, and all remaining strains, constituting the training dataset were flagged with a 1 using POPFLAG. The origin of each strain (chicken, ruminant or environment) was indicated using POPDATA.

Attribution tests were performed using all 583 animal or environmental isolates as well as the strains of interest for each attribution test (French clinical strains from different years of isolation and different origin). Strains belonging to the test dataset (clinical strains) were flagged with a 0, and all source strains, constituting the training dataset were flagged with a 1 (POPFLAG parameter). The origin of each strain (chicken, ruminants, environment or clinical) was indicated (POPDATA parameter).

Analyses were performed with 100,000 burn-in cycles followed by 100,000 MCMC repetitions with the parameters using source population information (USEPOPINFO parameter) with no admixture model assumed and allele frequency independent model. All analyses were repeated 10 times to insure the reproducibility of the attribution test. Average scores for each attribution were considered.

### Correction of attribution scores

The principle of correction made to the attribution is simple: it relies on the hypothesis that the errors FineStructure makes while self-attributing strains to a reservoir are also made when it attributes strains for which we don’t know the provenance. That means that if when we give the software 100 strains of chicken, 100 of ruminants, 100 of environment (these 3 populations being the training dataset), and 100 of human (being the ones we want to test), if it correctly self-attributes the 300 stains from the training dataset, there is no problem as far as we know with the learning, and the correction will have absolutely no effect on the attribution of the human cases. But if the software is wrongly attributing 20 of ruminant isolates, by attributing them to the chicken group, that means that part of the human isolates attributed to chicken will actually more likely be ruminants. The correction step does that and the calculations were built accordingly. The results from STRUCTURE can be viewed in the form of a matrix presenting the proportion of membership of each pre-defined population in each of the source clusters. The first three rows correspond to the 3 groups from the training dataset (chicken, ruminant and the environment); the following rows correspond to the test dataset (Supp. Fig. 3). In out-of-the-bag results, if the proportion of correctly self-attributed strains from chicken, ruminant and environment populations (respectively *C*_*C*_, *R*_*R*_ and *E*_*E*_) are below 0.9, there is a bias introduced in the proportion of clinical strains attributed to chicken, ruminant or environment (respectively *T*_*C*_, *T*_*R*_ and *T*_*E*_) due to the presence of samples in the training dataset that are similar to samples from another source. As this was the case here, a system of equations (Supp. Fig. 4) was implemented in order to correct this bias. Specifically, the proportion of strains wrongly attributed from the training dataset was used to estimate the proportion of strains wrongly attributed in the tested population.

This system was solved using an online equation solver (https://matrixcalc.org). Unbiased numbers of isolates (*T*_C_*, *T*_R_*and *T*_E_*) were then turned into proportions based on the number of isolates from the test dataset N.

## Supplementary information

Supplementary informations

## Data Availability

All 198 newly sequenced genomes were deposited in the Genbank and SRA public databases under the BioProject PRJNA497209.
