# Supplementary material for: Source attribution of Campylobacter jejuni shows variable importance of chicken and ruminants reservoirs in non-invasive and invasive French clinical isolates
Source: Sci Rep. 2019 May 30;9:8098. doi: 10.1038/s41598-019-44454-2 (PMC6542803; doi:10.1038/s41598-019-44454-2)
Supplement: Supplementary file 1 — Supplementary informations [file 41598_2019_44454_MOESM1_ESM.pdf]

Source attribution of *Campylobacter jejuni* shows variable importance of chicken and ruminants reservoirs in non-invasive and invasive French clinical isolates.

Elvire Berthenet, Amandine Thépault, Marianne Chemaly, Katell Rivoal, Astrid Ducournau[a], Alice Buissonnière[a], Lucie Bénéjat[a], Emilie Bessede[a], Francis Megraud[a,c], Samuel K Sheppard[d], Philippe Lehours[a,c] *

Supp. Figure 1. Attribution of French clinical isolates collected between 2009 and 2016 over time based on a collection. Attribution tests were performed using the STRUCTURE software with 10 replicates. Uncorrected proportion of isolates attributed to each reservoir (after model correction) is represented according to the year of isolation, with 95% CI. **A.** Attribution of French non-invasive clinical isolates collected in 2009 (n=39), 2015 (n=78) and 2016 (n=26). **B.** Attribution of French invasive clinical isolates collected in 2011 (n=17), 2012 (n=18), 2013 (n=33), 2014 (n=35), 2015 (n=33) and 2016 (n=37).

Supp. Figure 2. Map representing the geographic provenance of the 198 newly sequenced isolates. The color scale represents the number of strains isolated in each French department.

Supp. Figure 3. STRUCTURE out-of-the-bag results matrix. The main letter corresponds to the population from which the isolates were issued and the subscript corresponds to the population to which the isolates are attributed, with C, R, E and T respectively referring to chicken, ruminant, the environment and test populations.

Supp. Figure 4. System of 3 equations used to correct the attribution bias.

The main letter corresponds to the population from which the isolates were issued and the subscript corresponds to the population to which the isolates are attributed by Structure, with C, R, E and T respectively referring to chicken, ruminant, environment and test populations. N corresponds to the number of isolates in the test population. T_C* , T_R* and T_E* are the unknown variables corresponding to the unbiased number of isolates in the test population that are attributable to each of the sources.

Supp. Table 1: Details of the previously published 583 isolates from chicken, ruminant and environment reservoirs used in this study.

Supp. Table 2: Details of the previously published 118 French clinical isolates from 2009 and 2015 used in this study.

Supp. Table 3: Details of the 198 French clinical isolates sequenced in this study.

Supp. Table 4: List of the 15 host-segregating markers used for attribution in this study.

Suppl. Figure 1

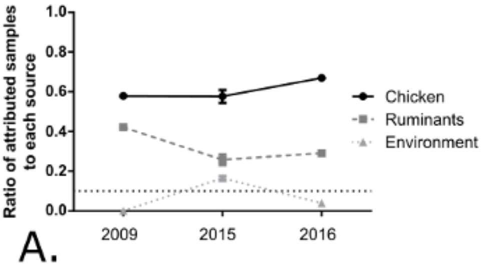

Suppl. Figure 2

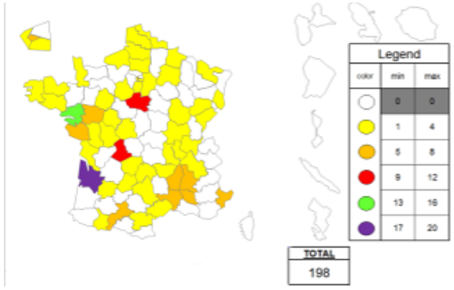

Suppl. Figure 3

$$\begin{matrix} C_C & C_R & C_E \\ R_C & R_R & R_E \\ E_C & E_R & E_E \\ T_C & T_R & T_E \end{matrix}$$

Suppl. Figure 4

$$\begin{cases} T_C \times N = C_C \times T_C{}^* + R_C \times T_R{}^* + E_C \times T_E{}^* - C_R \times T_C{}^* - C_E \times T_C{}^* \\ T_R \times N = R_R \times T_R{}^* + C_R \times T_C{}^* + E_R \times T_E{}^* - R_C \times T_R{}^* - R_E \times T_R{}^* \\ T_E \times N = E_E \times T_E{}^* + R_E \times T_R{}^* + C_E \times T_C{}^* - E_R \times T_E{}^* - E_C \times T_E{}^* \end{cases}$$

Supp. Table 1: Details of the previously published 583 isolates from chicken, ruminant and environment reservoirs used in this study

BIGSdb ID	Strain name	Country of isolation	Year of isolation	Isolation details	Source (for attribution)	genome size	number of contigs	MLST Sequence Type	MLST Clonal Complex
4	CAMP45	UK	2005	chicken offal or meat	Chicken	1596969	108	45	ST-45 complex
14	CAMP2381	New Zealand		environmental waters	Environment	1543877	324	2381	
22	CAMP2488	UK	2001	chicken	Chicken	1573736	169	257	ST-257 complex
39	chick2219	UK	2005	chicken offal or meat	Chicken	1616482	84	2219	ST-45 complex
40	chicka21	UK	2006	chicken offal or meat	Chicken	1726327	195	21	ST-21 complex
45	chick594	UK	2006	chicken offal or meat	Chicken	1609163	72	583	ST-45 complex
54	chick267	UK	2005	chicken offal or meat	Chicken	1591217	241	267	ST-283 complex
57	chick2213	UK	2005	chicken offal or meat	Chicken	1620325	158	334	ST-45 complex
63	chickc21	UK	2006	chicken offal or meat	Chicken	1679349	139	21	ST-21 complex
64	chick25	UK	2006	chicken offal or meat	Chicken	1698035	113	814	ST-661 complex
65	chick104	UK	2006	chicken offal or meat	Chicken	1761202	112	104	ST-21 complex
66	chick353	UK	2009	chicken	Chicken	1776210	129	353	ST-353 complex
67	chickb354	UK	2009	chicken	Chicken	1688706	143	354	ST-354 complex
68	chick573	UK	2009	chicken	Chicken	1838022	251	573	ST-573 complex
69	chick2568	UK	2009	chicken	Chicken	1821236	144	2568	ST-661 complex
70	chickc45	UK	2009	chicken	Chicken	1595762	343	45	ST-45 complex
71	chick19	UK	2009	chicken	Chicken	1689713	84	50	ST-21 complex
72	chick50	UK	2009	chicken	Chicken	1692341	63	50	ST-21 complex
73	chick53	UK	2009	chicken	Chicken	1651079	102	53	ST-21 complex
74	chick262	UK	2009	chicken	Chicken	1606379	65	262	ST-21 complex
75	chick266	UK	2009	chicken	Chicken	1693845	76	266	ST-21 complex
77	chick1086	UK	2009	chicken	Chicken	1692435	65	50	ST-21 complex
78	chick1360	UK	2009	chicken	Chicken	1693941	67	50	ST-21 complex
79	chick11	UK	2009	chicken	Chicken	1645238	120	11	ST-45 complex
80	chick137	UK	2009	chicken	Chicken	1734017	73	2030	ST-257 complex
81	chick1003	UK	2009	chicken	Chicken	1617200	106	1003	ST-45 complex
82	chick2048	UK	2009	chicken	Chicken	1631119	163	45	ST-45 complex
83	chick2197	UK	2009	chicken	Chicken	1667030	617	354	ST-354 complex
84	chick2223	UK	2009	chicken	Chicken	1605483	58	45	ST-45 complex
104	cow3214	UK	2003	cattle	Ruminants	1654338	115	45	ST-45 complex
105	chick354	UK	2004	chicken	Chicken	1697013	75	257	ST-257 complex
106	chick51	UK	2005	chicken	Chicken	1714044	58	51	ST-443 complex
107	chick1079	UK	2004	chicken	Chicken	1838061	273	1079	ST-573 complex
108	chick574	UK	2004	chicken	Chicken	1743461	80	574	ST-574 complex
109	chick814	UK	2004	chicken	Chicken	1759789	152	814	ST-661 complex
110	chickb21	UK	2003	chicken	Chicken	1656261	72	21	ST-21 complex
111	chickb45	UK	2004	chicken	Chicken	1649834	80	45	ST-45 complex
112	chickd45	UK	2004	chicken	Chicken	1618948	55	45	ST-45 complex
113	chick883	UK	2004	chicken	Chicken	1665144	72	883	ST-21 complex
114	chick230	UK	2004	chicken	Chicken	1633592	79	230	ST-45 complex
115	chick2663	UK	2006	chicken offal or meat	Chicken	3051265	7567	2663	ST-45 complex
122	starling177	UK		starling	Environment	1582720	52	177	ST-177 complex
123	starling682	UK		starling	Environment	4170275	714	682	ST-682 complex
124	starling45	UK		starling	Environment	1603669	112	45	ST-45 complex
125	starling1020	UK		starling	Environment	1578916	61	1020	ST-682 complex
126	goose1033	UK		goose	Environment	1663834	149	1033	ST-1034 complex
128	goose137	UK		goose	Environment	1600143	53	137	ST-45 complex
129	goose696	UK		goose	Environment	1561449	143	696	ST-1332 complex
130	duck702	UK		duck	Environment	1669953	108	702	ST-702 complex
131	duck45	UK		duck	Environment	1616162	60	45	ST-45 complex
172	Cj129-258	USA		cattle	Ruminants	1643841	98	459	ST-42 complex
173	Cj51494	USA		chicken	Chicken	1799590	195	4834	ST-353 complex
174	CjLMG23216	Belgium		chicken	Chicken	1708741	162	4835	
175	CjLMG23218	Belgium		chicken	Chicken	1677541	84	48	ST-48 complex
176	CjLMG23223	Belgium		chicken	Chicken	1701738	101	791	
177	CjLMG23263	Bosnia		chicken	Chicken	1739638	127	3504	ST-446 complex
178	Cj60004	USA		chicken	Chicken	1674182	108	4836	
180	CjLMG23269	Belgium		chicken	Chicken	1735582	151	4837	ST-353 complex
181	Cj55037	USA		chicken	Chicken	1598300	89	45	ST-45 complex
183	Cj86605	USA		chicken	Chicken	1637051	114	4840	ST-48 complex
185	CjATCC33560T	Belgium		cattle	Ruminants	1714676	221	403	ST-403 complex
187	Cj53161	USA		chicken	Chicken	1730741	145	4838	ST-353 complex
201	Cj51037	USA		chicken	Chicken	1747053	189	939	ST-353 complex
202	Cj110-21	USA		cattle	Ruminants	1615152	102	982	ST-21 complex
203	Cj87330	USA		chicken	Chicken	1618859	94	50	ST-21 complex
204	Cj87459	USA		chicken	Chicken	1772922	179	452	ST-353 complex
205	Cj140-16	USA		cattle	Ruminants	1677874	93	5161	ST-61 complex
206	Cj1213	USA		cattle	Ruminants	1673339	111	132	ST-508 complex
208	Cj1798	USA		cattle	Ruminants	1604776	94	61	ST-61 complex
209	Cj1854	USA		cattle	Ruminants	1619643	80	922	
210	Cj1893	USA		cattle	Ruminants	1700567	89	38	ST-48 complex
211	Cj1928	USA		cattle	Ruminants	1744799	174	806	ST-21 complex
213	Cj23210	Belgium		chicken	Chicken	1762534	117	380	
214	CjLMG23211	Belgium		chicken	Chicken	1669921	85	220	ST-179 complex
252	Cj_283	UK		chick	Chicken	1592046	113	267	ST-283 complex
269	13258	UK	1998	lamb offal or meat	Ruminants	1643890	37	48	ST-48 complex
271	13260	UK	1998	lamb offal or meat	Ruminants	2096004	906		
273	13262	UK	1994	sand (bathing beach)	Environment	1619347	30	177	ST-177 complex
278	SS_060	UK	2008	carcass swab	Chicken	1771216	99	4468	ST-661 complex
280	SS_068	UK	2008	carcass swab	Chicken	1781971	99	449	
281	SS_069	UK	2008	carcass swab	Chicken	1765861	70	574	ST-574 complex
283	SS_077	UK	2008	carcass swab	Chicken	1643818	30	48	ST-48 complex
285	SS_092	UK	2008	carcass swab	Chicken	1740859	62	353	ST-353 complex
286	SS_121	UK	2008	carcass swab	Chicken	1742151	27	2030	ST-257 complex
288	SS_133	UK	2008	carcass swab	Chicken	1682531	46	137	ST-45 complex
290	SS_153	UK	2008	carcass swab	Chicken	1659745	36	21	ST-21 complex
295	SS_174	UK	2008	carcass swab	Chicken	1805299	93	3895	ST-353 complex
298	SS_199	UK	2008	carcass swab	Chicken	1625592	40	4454	
299	SS_202	UK	2008	carcass swab	Chicken	1690291	41	257	ST-257 complex
300	SS_204	UK	2008	carcass swab	Chicken	1710609	49	354	ST-354 complex
301	SS_208	UK	2008	carcass swab	Chicken	1712243	101	464	ST-464 complex
302	SS_210	UK	2008	carcass swab	Chicken	1778211	106	1489	ST-354 complex
303	SS_214	UK	2008	carcass swab	Chicken	1694418	43	48	ST-48 complex
304	SS_216	UK	2008	carcass swab	Chicken	1668434	33	51	ST-443 complex
306	SS_223	UK	2008	carcass swab	Chicken	1692291	44	702	ST-702 complex
308	SS_242	UK	2008	carcass swab	Chicken	1733234	104	354	ST-354 complex
309	SS_249	UK	2008	carcass swab	Chicken	1698107	40	45	ST-45 complex
311	SS_256	UK	2008	carcass swab	Chicken	1747071	35	257	ST-257 complex
312	SS_257	UK	2008	carcass swab	Chicken	1695658	35	583	ST-45 complex
315	SS_300	UK	2008	carcass swab	Chicken	1833875	137	573	ST-573 complex
317	SS_307	UK	2008	carcass swab	Chicken	1645273	39	775	ST-52 complex
320	SS_313	UK	2008	carcass swab	Chicken	1657482	53	19	ST-21 complex
321	SS_315	UK	2008	carcass swab	Chicken	1651099	40	45	ST-45 complex

Supp. Table 1: Details of the previously published 583 isolates from chicken, ruminant and environment reservoirs used in this study

BIGSdb ID	Strain name	Country of isolation	Year of isolation	Isolation details	Source (for attribution)	genome size	number of contigs	MLST Sequence Type	MLST Clonal Complex
323	SS_320	UK	2008	carcass swab	Chicken	1806828	77	607	ST-607 complex
324	SS_322	UK	2008	carcass swab	Chicken	1618172	51	45	ST-45 complex
325	SS_002	UK	2006	cattle	Ruminants	1661910	47	19	ST-21 complex
329	SS_063	UK	2008	caecal sample	Chicken	3325215	78	2314	ST-1034 complex
330	SS_065	UK	2008	caecal sample	Chicken	1737207	28	2030	ST-257 complex
331	SS_066	UK	2008	caecal sample	Chicken	1747928	93	2135	ST-21 complex
334	SS_071	UK	2009	caecal sample	Chicken	1644938	29	267	ST-283 complex
336	SS_079	UK	2008	caecal sample	Chicken	1746301	36	2030	ST-257 complex
337	SS_081	UK	2008	caecal sample	Chicken	1741006	45	574	ST-574 complex
339	SS_084	UK	2007	caecal sample	Chicken	1689974	42	775	ST-52 complex
340	SS_086	UK	2009	caecal sample	Chicken	1791276	35	257	ST-257 complex
342	SS_091	UK	2009	caecal sample	Chicken	1653604	35	583	ST-45 complex
344	SS_100	UK	2009	caecal sample	Chicken	1773941	80	3895	ST-353 complex
345	SS_105	UK	2008	caecal sample	Chicken	1780889	94	3009	ST-433 complex
347	SS_107	UK	2008	caecal sample	Chicken	1697909	37	48	ST-48 complex
348	SS_110	UK	2009	caecal sample	Chicken	1746714	58	464	ST-464 complex
349	SS_113	UK	2009	caecal sample	Chicken	1922380	204	573	ST-573 complex
351	SS_116	UK	2009	caecal sample	Chicken	1737179	55	2135	ST-21 complex
355	SS_123	UK	2009	caecal sample	Chicken	1700988	27	257	ST-257 complex
356	SS_128	UK	2007	caecal sample	Chicken	1829462	1260		
357	SS_131	UK	2008	caecal sample	Chicken	1748880	74	4460	ST-661 complex
359	SS_139	UK	2008	caecal sample	Chicken	1680381	53	50	ST-21 complex
360	SS_141	UK	2008	caecal sample	Chicken	1834130	122	573	ST-573 complex
364	SS_147	UK	2009	caecal sample	Chicken	1825040	135	863	ST-607 complex
365	SS_149	UK	2007	caecal sample	Chicken	1630222	49	2314	ST-1034 complex
367	SS_152	UK	2009	caecal sample	Chicken	1804023	63	2786	ST-661 complex
368	SS_154	UK	2009	caecal sample	Chicken	1656094	46	48	ST-48 complex
369	SS_158	UK	2007	caecal sample	Chicken	1746626	80	1408	ST-433 complex
370	SS_167	UK	2009	caecal sample	Chicken	1745279	42	21	ST-21 complex
371	SS_169	UK	2009	caecal sample	Chicken	1612209	26	137	ST-45 complex
372	SS_170	UK	2008	caecal sample	Chicken	1800990	57	4452	
373	SS_171	UK	2009	caecal sample	Chicken	1732408	79	2314	ST-1034 complex
374	SS_172	UK	2009	caecal sample	Chicken	1793591	140	353	ST-353 complex
375	SS_173	UK	2009	caecal sample	Chicken	1741628	43	574	ST-574 complex
376	SS_175	UK	2008	caecal sample	Chicken	1699112	36	21	ST-21 complex
378	SS_177	UK	2008	caecal sample	Chicken	1634128	36	137	ST-45 complex
379	SS_178	UK	2008	caecal sample	Chicken	1659926	34	21	ST-21 complex
380	SS_179	UK	2008	caecal sample	Chicken	1692440	44	702	ST-702 complex
381	SS_180	UK	2009	caecal sample	Chicken	1778947	105	449	
382	SS_185	UK	2009	caecal sample	Chicken	1619030	58	11	ST-45 complex
384	SS_187	UK	2009	caecal sample	Chicken	1738767	39	50	ST-21 complex
385	SS_188	UK	2009	caecal sample	Chicken	1741466	34	2030	ST-257 complex
387	SS_200	UK	2008	caecal sample	Chicken	1779885	67	464	ST-464 complex
388	SS_203	UK	2008	caecal sample	Chicken	1614608	51	4454	
389	SS_206	UK	2008	caecal sample	Chicken	1769559	80	4468	ST-661 complex
390	SS_212	UK	2009	caecal sample	Chicken	1799379	122	1489	ST-354 complex
392	SS_218	UK	2009	caecal sample	Chicken	1654821	48	45	ST-45 complex
393	SS_220	UK	2009	caecal sample	Chicken	1743268	39	257	ST-257 complex
394	SS_225	UK	2007	caecal sample	Chicken	1773682	59	353	ST-353 complex
395	SS_226	UK	2009	caecal sample	Chicken	1644140	36	51	ST-443 complex
396	SS_227	UK	2008	caecal sample	Chicken	1786340	122	1489	ST-354 complex
397	SS_231	UK	2009	caecal sample	Chicken	1717635	65	4432	ST-661 complex
399	SS_235	UK	2009	caecal sample	Chicken	1621197	45	2314	ST-1034 complex
401	SS_243	UK	2008	caecal sample	Chicken	1728519	88	4472	ST-702 complex
405	SS_252	UK	2007	caecal sample	Chicken	1628765	43	2197	ST-45 complex
406	SS_254	UK	2008	caecal sample	Chicken	1694508	37	583	ST-45 complex
409	SS_259	UK	2007	caecal sample	Chicken	1703755	44	354	ST-354 complex
411	SS_262	UK	2008	caecal sample	Chicken	1741660	52	574	ST-574 complex
412	SS_287	UK	2008	caecal sample	Chicken	1792093	48	574	ST-574 complex
414	SS_289	UK	2008	caecal sample	Chicken	1714472	26	51	ST-443 complex
416	SS_295	UK	2008	caecal sample	Chicken	1682408	44	45	ST-45 complex
417	SS_296	UK	2007	caecal sample	Chicken	1741436	31	2030	ST-257 complex
418	SS_297	UK	2007	caecal sample	Chicken	1694233	34	50	ST-21 complex
421	SS_301	UK	2008	caecal sample	Chicken	1620834	56	19	ST-21 complex
422	SS_302	UK	2008	caecal sample	Chicken	1644997	38	775	ST-52 complex
423	SS_303	UK	2007	caecal sample	Chicken	1689736	48	19	ST-21 complex
424	SS_304	UK	2008	caecal sample	Chicken	1747485	33	257	ST-257 complex
427	SS_310	UK	2007	caecal sample	Chicken	1780790	112	574	ST-574 complex
428	SS_312	UK	2008	caecal sample	Chicken	1741331	38	257	ST-257 complex
429	SS_314	UK	2008	caecal sample	Chicken	1629376	36	2197	ST-45 complex
430	SS_317	UK	2008	caecal sample	Chicken	1764732	70	2568	ST-661 complex
433	SS_005	UK	2010	chicken	Chicken	1735616	39	2030	ST-257 complex
434	SS_006	UK	2011	chicken	Chicken	1608621	42	45	ST-45 complex
435	SS_025	UK	2005	chicken offal or meat	Chicken	1694216	29	257	ST-257 complex
437	SS_027	UK	2005	chicken offal or meat	Chicken	1700062	31	257	ST-257 complex
438	SS_030	UK	2005	chicken offal or meat	Chicken	1655562	31	48	ST-48 complex
440	SS_032	UK	2005	chicken offal or meat	Chicken	1601151	17	137	ST-45 complex
441	SS_033	UK	2005	chicken offal or meat	Chicken	1694059	27	257	ST-257 complex
443	SS_036	UK	2005	chicken offal or meat	Chicken	1615431	41	25	ST-45 complex
444	SS_037	UK	2005	chicken offal or meat	Chicken	1742468	33	257	ST-257 complex
445	SS_038	UK	2005	chicken offal or meat	Chicken	1808275	447	257	ST-257 complex
446	SS_039	UK	2005	chicken offal or meat	Chicken	1608730	49	233	ST-45 complex
447	SS_040	UK	2005	chicken offal or meat	Chicken	1618219	69	45	ST-45 complex
448	SS_041	UK	2005	chicken offal or meat	Chicken	1639891	38	661	ST-661 complex
449	SS_042	UK	2005	chicken offal or meat	Chicken	1615661	50	25	ST-45 complex
451	SS_044	UK	2005	chicken offal or meat	Chicken	1601327	34	267	ST-283 complex
452	SS_045	UK	2005	chicken offal or meat	Chicken	1694469	30	257	ST-257 complex
453	SS_046	UK	2005	chicken offal or meat	Chicken	1601894	24	137	ST-45 complex
458	SS_053	UK	2005	chicken offal or meat	Chicken	1614159	43	25	ST-45 complex
460	SS_055	UK	2005	chicken offal or meat	Chicken	1778525	26	257	ST-257 complex
461	SS_056	UK	2005	chicken offal or meat	Chicken	1767904	65	607	ST-607 complex
462	SS_057	UK	2005	chicken offal or meat	Chicken	1731351	31	48	ST-48 complex
463	SS_058	UK	2005	chicken offal or meat	Chicken	1630422	34	45	ST-45 complex
466	SS_278	UK	2005	chicken offal or meat	Chicken	1707667	37	50	ST-21 complex
467	SS_280	UK	2005	chicken offal or meat	Chicken	1682909	47	45	ST-45 complex

Supp. Table 1: Details of the previously published 583 isolates from chicken, ruminant and environment reservoirs used in this study

BIGSdb ID	Strain name	Country of isolation	Year of isolation	Isolation details	Source (for attribution)	genome size	number of contigs	MLST Sequence Type	MLST Clonal Complex
469	SS_282	UK	2005	chicken offal or meat	Chicken	1739304	30	574	ST-574 complex
470	SS_283	UK	2005	chicken offal or meat	Chicken	1740806	61	354	ST-354 complex
471	SS_072	UK	2008	farm environment	Chicken	1626459	30	2314	ST-1034 complex
472	SS_075	UK	2008	farm environment	Chicken	1644564	42	45	ST-45 complex
474	SS_078	UK	2008	farm environment	Chicken	1907153	162	3965	ST-573 complex
475	SS_083	UK	2008	farm environment	Chicken	1826522	99	573	ST-573 complex
476	SS_085	UK	2008	farm environment	Chicken	1701988	42	354	ST-354 complex
477	SS_088	UK	2008	farm environment	Chicken	1702287	43	354	ST-354 complex
478	SS_089	UK	2008	farm environment	Chicken	1623863	33	2314	ST-1034 complex
479	SS_094	UK	2008	farm environment	Chicken	1774824	94	3895	ST-353 complex
480	SS_095	UK	2008	farm environment	Chicken	1760469	867		
483	SS_098	UK	2008	farm environment	Chicken	1645963	37	48	ST-48 complex
484	SS_099	UK	2008	farm environment	Chicken	1642422	35	51	ST-443 complex
486	SS_102	UK	2008	farm environment	Chicken	1730174	88	2568	ST-661 complex
488	SS_104	UK	2008	farm environment	Chicken	1623834	32	2314	ST-1034 complex
489	SS_108	UK	2008	farm environment	Chicken	1692592	44	50	ST-21 complex
490	SS_109	UK	2008	farm environment	Chicken	1695446	42	50	ST-21 complex
491	SS_111	UK	2008	farm environment	Chicken	1728471	103	2568	ST-661 complex
492	SS_112	UK	2008	farm environment	Chicken	1842718	106	1910	ST-353 complex
494	SS_117	UK	2008	farm environment	Chicken	1708046	44	574	ST-574 complex
495	SS_120	UK	2008	farm environment	Chicken	1610967	38	3573	
496	SS_126	UK	2008	farm environment	Chicken	1794724	141	449	
497	SS_129	UK	2008	farm environment	Chicken	1715416	52	354	ST-354 complex
498	SS_130	UK	2008	farm environment	Chicken	1778520	77	3895	ST-353 complex
499	SS_134	UK	2008	farm environment	Chicken	1610925	43	3573	
500	SS_135	UK	2008	farm environment	Chicken	1922989	227	3965	ST-573 complex
1699	SS_136	UK	2008	farm environment	Chicken	1703724	43	354	ST-354 complex
1700	SS_137	UK	2008	farm environment	Chicken	1622039	34	2314	ST-1034 complex
1701	SS_138	UK	2008	farm environment	Chicken	1610429	37	3573	
1702	SS_140	UK	2008	farm environment	Chicken	1606112	33	45	ST-45 complex
1704	SS_144	UK	2008	farm environment	Chicken	1610072	35	3573	
1705	SS_148	UK	2008	farm environment	Chicken	1700593	27	257	ST-257 complex
1706	SS_155	UK	2008	farm environment	Chicken	1609660	36	3573	
1707	SS_156	UK	2008	farm environment	Chicken	1696216	48	50	ST-21 complex
1709	SS_162	UK	2008	farm environment	Chicken	1621240	32	2314	ST-1034 complex
1710	SS_163	UK	2008	farm environment	Chicken	1827089	82	2568	ST-661 complex
1711	SS_164	UK	2008	farm environment	Chicken	1609962	34	3573	
1712	SS_165	UK	2008	farm environment	Chicken	1845722	115	1910	ST-353 complex
1713	SS_166	UK	2008	farm environment	Chicken	1780815	90	3895	ST-353 complex
1714	SS_181	UK	2008	farm environment	Chicken	1695542	43	50	ST-21 complex
1715	SS_182	UK	2008	farm environment	Chicken	1815088	69	2568	ST-661 complex
1717	SS_184	UK	2008	farm environment	Chicken	1613283	44	3573	
1718	SS_190	UK	2008	farm environment	Chicken	1683758	76	775	ST-52 complex
1719	SS_191	UK	2008	farm environment	Chicken	1693519	43	50	ST-21 complex
1720	SS_195	UK	2008	farm environment	Chicken	1856991	133	573	ST-573 complex
1721	SS_196	UK	2008	farm environment	Chicken	1855217	158	573	ST-573 complex
1722	SS_197	UK	2008	farm environment	Chicken	1631280	33	2197	ST-45 complex
1723	SS_198	UK	2008	farm environment	Chicken	1629287	32	2197	ST-45 complex
1724	SS_201	UK	2008	farm environment	Chicken	1821471	62	2568	ST-661 complex
1726	SS_207	UK	2008	farm environment	Chicken	1807750	78	607	ST-607 complex
1727	SS_209	UK	2008	farm environment	Chicken	1691280	424	45	ST-45 complex
1728	SS_215	UK	2008	farm environment	Chicken	1610761	37	3573	
1729	SS_217	UK	2008	farm environment	Chicken	1667520	572	2314	ST-1034 complex
1730	SS_221	UK	2008	farm environment	Chicken	1826926	75	2568	ST-661 complex
1731	SS_222	UK	2008	farm environment	Chicken	1696365	44	50	ST-21 complex
1732	SS_228	UK	2008	farm environment	Chicken	1828902	130	2568	ST-661 complex
1733	SS_230	UK	2008	farm environment	Chicken	1704240	539	45	ST-45 complex
1734	SS_232	UK	2008	farm environment	Chicken	1871542	157	1910	ST-353 complex
1735	SS_233	UK	2008	farm environment	Chicken	1835388	215		
1737	SS_237	UK	2008	farm environment	Chicken	1660604	75	45	ST-45 complex
1738	SS_238	UK	2008	farm environment	Chicken	1630210	36	2197	ST-45 complex
1739	SS_239	UK	2008	farm environment	Chicken	1625540	39	2314	ST-1034 complex
1740	SS_244	UK	2008	farm environment	Chicken	1650051	55	11	ST-45 complex
1756	SS_004	UK	2010	sheep	Ruminants	1705466	32	206	ST-206 complex
1762	SS_008	UK	2006	chicken	Chicken	1642326	37	51	ST-443 complex
1763	SS_009	UK	2006	cattle	Ruminants	1588042	34	42	ST-42 complex
1768	SS_015	UK	2006	cattle	Ruminants	1689703	22	61	ST-61 complex
1771	SS_020	UK	2006	chicken	Chicken	1645679	34	45	ST-45 complex
1774	SS_024	UK	2005	carcass swab	Chicken	1747830	44	354	ST-354 complex
1775	SS_028	UK	2005	carcass swab	Chicken	1631165	41	583	ST-45 complex
1776	SS_029	UK	2005	carcass swab	Chicken	1654669	42	45	ST-45 complex
1780	SS_324	UK	2006	sheep	Ruminants	1667948	28	206	ST-206 complex
1782	SS_327	UK	2006	cattle	Ruminants	1752712	66	48	ST-48 complex
1785	SS_330	UK	2006	sheep	Ruminants	1667637	46	21	ST-21 complex
1786	SS_332	UK	2006	sheep	Ruminants	1653346	43	53	ST-21 complex
1787	SS_333	UK	2006	sheep	Ruminants	1657495	44	19	ST-21 complex
1791	SS_337	UK	2009	chicken	Chicken	1731853	34	5	ST-353 complex
1793	SS_339	UK	2011	chicken	Chicken	1768201	57	354	ST-354 complex
1794	SS_340	UK	2011	chicken	Chicken	1696209	27	257	ST-257 complex
1795	SS_341	UK	2011	chicken	Chicken	1754667	104	574	ST-574 complex
1796	SS_342	UK	2010	cattle	Ruminants	1628871	41		
1799	SS_345	UK	2005	carcass swab	Chicken	1757914	33	51	ST-443 complex
1800	SS_375	UK	2007	chicken	Chicken	1651291	29	45	ST-45 complex
1801	SS_376	UK	2007	chicken	Chicken	1842527	529		
1802	SS_377	UK	2007	chicken	Chicken	1734164	96	2135	ST-21 complex
1803	SS_378	UK	2007	chicken	Chicken	1732695	94	2568	ST-661 complex
1804	SS_379	UK	2007	chicken	Chicken	1815693	36	52	ST-52 complex
1805	SS_380	UK	2007	chicken	Chicken	1794937	48	863	ST-607 complex
1806	SS_381	UK	2007	chicken	Chicken	1645659	25	48	ST-48 complex
1807	SS_382	UK	2007	chicken	Chicken	1841270	218	4415	
1808	SS_383	UK	2007	chicken	Chicken	1595910	31	267	ST-283 complex
1810	SS_385	UK	2007	chicken	Chicken	1873508	103	573	ST-573 complex
1811	SS_386	UK	2007	chicken	Chicken	1641190	39	51	ST-443 complex
1812	SS_387	UK	2007	chicken	Chicken	1747152	31	257	ST-257 complex
1814	SS_389	UK	2007	chicken	Chicken	1672069	50	702	ST-702 complex
1817	SS_392	UK	2007	chicken	Chicken	1609289	26	583	ST-45 complex
1818	SS_393	UK	2007	chicken	Chicken	1769720	51	607	ST-607 complex
1819	SS_394	UK	2007	chicken	Chicken	1763243	87	1491	
1822	SS_397	UK	2007	chicken	Chicken	1664293	46	21	ST-21 complex
1829	SS_404	UK	2008	chicken	Chicken	1740934	30	257	ST-257 complex
1830	SS_405	UK	2008	carcass swab	Chicken	1888253	191	573	ST-573 complex
1831	SS_406	UK	2008	carcass swab	Chicken	1732626	29	257	ST-257 complex
1832	SS_407	UK	2008	chicken	Chicken	1620665	37	1709	ST-1034 complex
1833	SS_408	UK	2008	farm environment	Chicken	1751014	64	464	ST-464 complex
1834	SS_410	UK	2008	carcass swab	Chicken	1751989	61	4460	ST-661 complex
1835	SS_411	UK		carcass swab	Chicken	1600320	25	137	ST-45 complex
2255	B2	Canada		cattle	Ruminants	1657208	358	61	ST-61 complex
2256	B3	Canada		cattle	Ruminants	1649613	85	21	ST-21 complex
2257	B4	Canada		cattle	Ruminants	1662784	98	21	ST-21 complex

Supp. Table 1: Details of the previously published 583 isolates from chicken, ruminant and environment reservoirs used in this study

BIGSdb ID	Strain name	Country of isolation	Year of isolation	Isolation details	Source (for attribution)	genome size	number of contigs	MLST Sequence Type	MLST Clonal Complex
2258	C1	Canada		chicken	Chicken	1646990	87	45	ST-45 complex
2259	C10	Canada		chicken	Chicken	1877206	165	924	ST-607 complex
2260	C11	Canada		chicken	Chicken	5182860	1538	48	ST-48 complex
2261	C12	Canada		chicken	Chicken	1726897	93	48	ST-48 complex
2262	C13	Canada		chicken	Chicken	1671504	110	982	ST-21 complex
2265	C16	Canada		chicken	Chicken	1651277	23	45	ST-45 complex
2266	C2	Canada		chicken	Chicken	1714446	482	45	ST-45 complex
2267	C3	Canada		chicken	Chicken	1792487	86	460	ST-460 complex
2268	C4	Canada		chicken	Chicken	1791666	89	460	ST-460 complex
2269	C5	Canada		chicken	Chicken	1787445	105	460	ST-460 complex
2270	C6	Canada		chicken	Chicken	1698047	96	929	ST-257 complex
2271	C7	Canada		chicken	Chicken	1681502	183	929	ST-257 complex
2272	C8	Canada		chicken	Chicken	1878254	165	924	ST-607 complex
2273	C9	Canada		chicken	Chicken	1875746	187		
2313	S1	Canada		sediment isolate	Environment	1761270	123	4186	ST-179 complex
2314	S2	Canada		sediment isolate	Environment	1626772	219	45	ST-45 complex
2315	S3	Canada		sediment isolate	Environment	1644689	71	45	ST-45 complex
2317	S5	Canada		sediment isolate	Environment	1641049	51	538	ST-45 complex
2318	S6	Canada		sediment isolate	Environment	1639161	59	45	ST-45 complex
2323	W10	Canada		water isolate; drain	Environment	1645756	62	61	ST-61 complex
2324	W11	Canada		water isolate; drain	Environment	1618784	71	61	ST-61 complex
2325	W12	Canada		water isolate; river	Environment	1702139	113	929	ST-257 complex
2326	W13	Canada		water isolate; river	Environment	1691851	235	693	
2327	W14	Canada		water isolate; river	Environment	1619889	90	8	ST-21 complex
2328	W15	Canada		water isolate; river	Environment	1689029	169	8	ST-21 complex
2329	W16	Canada		water isolate	Environment	1652712	99	991	ST-692 complex
2330	W17	Canada		water isolate; drain	Environment	1574127	451	8	ST-21 complex
2331	W18	Canada		water isolate; drain	Environment	1683259	65	929	ST-257 complex
2332	W19	Canada		water isolate	Environment	1681232	807		
2333	W2	Canada		water isolate	Environment	1654462	111	459	ST-42 complex
2334	W20	Canada		water isolate;river	Environment	1574911	84	991	ST-692 complex
2335	W21	Canada		water isolate; river	Environment	2170358	3966		
2336	W22	Canada		water isolate	Environment	1625935	13	45	ST-45 complex
2338	W24	Canada		water isolate; river	Environment	1572033	343	45	ST-45 complex
2339	W25	Canada		water isolate	Environment	1677609	121	9353	ST-1034 complex
2341	W27	Canada		water isolate	Environment	1595906	68	267	ST-283 complex
2342	W28	Canada		water isolate	Environment	1582275	123	991	ST-692 complex
2343	W29	Canada		water isolate	Environment	1635028	55	538	ST-45 complex
2345	W30	Canada		water isolate	Environment	1717445	8	929	ST-257 complex
2346	W31	Canada		water isolate	Environment	1641153	59	538	ST-45 complex
2347	W32	Canada		water isolate	Environment	1596577	73	267	ST-283 complex
2348	W33	Canada		water isolate	Environment	1770563	117	4186	ST-179 complex
2349	W34	Canada		water isolate	Environment	1640068	60	45	ST-45 complex
2350	W35	Canada		water isolate	Environment	1596075	64	267	ST-283 complex
2351	W36	Canada		water isolate	Environment	1593995	116	267	ST-283 complex
2352	W37	Canada		water isolate	Environment	1595372	55	267	ST-283 complex
2353	W38	Canada		water isolate	Environment	1595133	58	267	ST-283 complex
2354	W39	Canada		water isolate	Environment	6395816	887	45	ST-45 complex
2355	W4	Canada		water isolate	Environment	1618140	168	45	ST-45 complex
2356	W40	Canada		water isolate	Environment	1807759	109	4186	ST-179 complex
2357	W41	Canada		water isolate	Environment	1651139	65	459	ST-42 complex
2358	W42	Canada		water isolate	Environment	1601684	73	459	ST-42 complex
2359	W43	Canada		water isolate	Environment	1460606	316		
2360	W44	Canada		water isolate	Environment	1776086	110	806	ST-21 complex
2361	W45	Canada		water isolate	Environment	1650450	71	459	ST-42 complex
2362	W46	Canada		water isolate	Environment	1811036	111	933	ST-403 complex
2364	W6	Canada		water isolate	Environment	1797600	410	9355	
2367	W9	Canada		water isolate	Environment	1619385	14	45	ST-45 complex
2469	RM9357	USA	2012	cattle	Ruminants	1673384	65		
2470	RM9168	USA	2012	cattle	Ruminants	1729552	69	21	ST-21 complex
2471	RM8893	USA	2012	cattle	Ruminants	1693259	62	8	ST-21 complex
2472	RM8004	USA	2012	cattle	Ruminants	1837933	62	806	ST-21 complex
2475	RM10985	USA	2012	cattle	Ruminants	1626077	64	21	ST-21 complex
2476	D42a	USA	2005	chicken	Chicken	1609668	1847	21	ST-21 complex
2479	RM12500	USA	2012	cattle	Ruminants	1614196	43	61	ST-61 complex
3004	SS_1029	UK		chicken	Chicken	1614153	20	61	ST-61 complex
3007	SS_1034	The Netherlands		cattle	Ruminants	1614646	29	60	ST-61 complex
3008	SS_1035	UK		cattle	Ruminants	1651287	22	61	ST-61 complex
3009	SS_1036	UK		cattle	Ruminants	1651552	27	61	ST-61 complex
3011	SS_1040	UK		cattle	Ruminants	1697581	19	61	ST-61 complex
3012	SS_1045	UK		cattle	Ruminants	5920652	245		
3014	SS_1050	UK		cattle	Ruminants	5918138	202	61	ST-61 complex
3016	SS_1052	UK		cattle	Ruminants	5873814	101	864	ST-61 complex
3018	SS_1061	UK		cattle	Ruminants	1651639	21	61	ST-61 complex
3019	SS_1062	UK		sheep	Ruminants	1695040	138	61	ST-61 complex
3020	SS_1063	UK		cattle	Ruminants	1614194	20	61	ST-61 complex
3021	SS_1064	UK		cattle	Ruminants	1701947	28	61	ST-61 complex
3023	SS_1067	UK		cattle	Ruminants	2067535	1450	820	ST-61 complex
3024	SS_1069	UK		sheep	Ruminants	1661461	47	61	ST-61 complex
3025	SS_1070	UK		cattle	Ruminants	1744460	37	61	ST-61 complex
3027	SS_1072	UK		sheep	Ruminants	1680544	73	61	ST-61 complex
3032	SS_975	N/A	2001	wild bird	Environment	1620699	66	1342	
4713	AC0264	France		caecal sample	Chicken	1659041	33	19	ST-21 complex
4714	AC0281	France		carcass swab	Chicken	1658623	31	19	ST-21 complex
4715	AC0302	France		caecal sample	Chicken	1708711	43	290	ST-206 complex
4716	AC0400	France		carcass swab	Chicken	1613836	33	45	ST-45 complex
4717	AC0465	France		carcass swab	Chicken	1609517	46	1964	ST-45 complex
4718	AC0473	France		carcass swab	Chicken	1687124	73	21	ST-21 complex
4719	AC0559	France		caecal sample	Chicken	1680321	52	475	ST-48 complex
4720	AC0568	France		carcass swab	Chicken	1661457	78	21	ST-21 complex
4721	AC0691	France		carcass swab	Chicken	1632863	59	2197	ST-45 complex
4722	AC0745	France		carcass swab	Chicken	1739167	256	50	ST-21 complex
4723	AC0791	France		caecal sample	Chicken	1687432	47	5904	ST-48 complex
4724	AC0927	France		carcass swab	Chicken	1824644	105	400	ST-353 complex
4725	AC0994	France		carcass swab	Chicken	1702265	62	21	ST-21 complex
4726	AC1136	France		carcass swab	Chicken	1659757	33	19	ST-21 complex
4727	AC1151	France		carcass swab	Chicken	1691809	35	883	ST-21 complex
4728	AC1363	France		carcass swab	Chicken	1710993	25	122	ST-206 complex
4729	AC1474	France		carcass swab	Chicken	1733621	312	19	ST-21 complex
4730	AC1476	France		carcass swab	Chicken	1651196	61	19	ST-21 complex
4731	AC2327	France		carcass swab	Chicken	1654077	60	572	ST-206 complex
4732	AC2811	France		caecal sample	Chicken	1700937	74	21	ST-21 complex
4733	AC2823	France		carcass swab	Chicken	1656286	51	19	ST-21 complex
4734	AC3107	France		caecal sample	Chicken	1654114	51	19	ST-21 complex
4735	AC3201	France		caecal sample	Chicken	1664084	82	50	ST-21 complex
4736	AC3497	France		caecal sample	Chicken	1658541	66	19	ST-21 complex
4737	AC3940	France		carcass swab	Chicken	1721782	56	221	ST-206 complex
4738	AC4322	France		caecal sample	Chicken	1747849	79	4823	ST-464 complex
4739	AC4401	France		carcass swab	Chicken	1684571	53	6360	ST-353 complex
4740	AC4520	France		carcass swab	Chicken	1643378	319	475	ST-48 complex
4741	AC4896	France		carcass swab	Chicken	1652363	151	48	ST-48 complex

Supp. Table 1: Details of the previously published 583 isolates from chicken, ruminant and environment reservoirs used in this study

BIGSdb ID	Strain name	Country of isolation	Year of isolation	Isolation details	Source (for attribution)	genome size	number of contigs	MLST Sequence Type	MLST Clonal Complex
4742	C0113	France		chicken offal or meat	Chicken	1612413	87	45	ST-45 complex
4743	C0118	France		chicken offal or meat	Chicken	1814140	135	21	ST-21 complex
4744	C0185	France		chicken offal or meat	Chicken	1726094	42	19	ST-21 complex
4745	C0191	France		chicken offal or meat	Chicken	1766863	81	464	ST-464 complex
4746	C0230	France		chicken offal or meat	Chicken	1692448	55	47	ST-21 complex
4747	C0274	France		chicken offal or meat	Chicken	1654800	107	19	ST-21 complex
4748	C0276	France		chicken offal or meat	Chicken	1661133	38	19	ST-21 complex
4749	C0303	France		chicken offal or meat	Chicken	1638821	65	475	ST-48 complex
4750	C0402	France		chicken offal or meat	Chicken	1689777	89	19	ST-21 complex
4751	C0519	France		chicken offal or meat	Chicken	1728037	115	19	ST-21 complex
4752	C0531	France		chicken offal or meat	Chicken	1704822	49	3547	ST-206 complex
4753	C0743	France		chicken offal or meat	Chicken	1691598	50	48	ST-48 complex
4754	C0757	France		chicken offal or meat	Chicken	1758282	89	2882	ST-353 complex
4755	C0784	France		chicken offal or meat	Chicken	1664186	89	3633	ST-21 complex
4756	C0848	France		chicken offal or meat	Chicken	1601808	29	137	ST-45 complex
4757	C1055	France		chicken offal or meat	Chicken	1604923	34	334	ST-45 complex
4758	C1061	France		chicken offal or meat	Chicken	1621098	37	19	ST-21 complex
4759	C1141	France		chicken offal or meat	Chicken	1627507	87	19	ST-21 complex
4760	C1251	France		chicken offal or meat	Chicken	1740716	85	21	ST-21 complex
4761	C1267	France		chicken offal or meat	Chicken	1712634	79	21	ST-21 complex
4762	C1387	France		chicken offal or meat	Chicken	1811687	82	19	ST-21 complex
4763	C1516	France		chicken offal or meat	Chicken	1657895	49	19	ST-21 complex
4764	C1656	France		chicken offal or meat	Chicken	1735651	47	400	ST-353 complex
4765	C1658	France		chicken offal or meat	Chicken	1795737	87	464	ST-464 complex
4767	C1863	France		chicken offal or meat	Chicken	1681655	31		
4768	C1871	France		chicken offal or meat	Chicken	1663035	43	21	ST-21 complex
4769	C2094	France		chicken offal or meat	Chicken	1732572	50	5138	ST-206 complex
4770	C2122	France		chicken offal or meat	Chicken	1826719	395	356	ST-353 complex
4771	C2228	France		chicken offal or meat	Chicken	1769975	52		
4772	C2234	France		chicken offal or meat	Chicken	1713672	39	21	ST-21 complex
4773	C2240	France		chicken offal or meat	Chicken	1645799	39	45	ST-45 complex
4774	C2244	France		chicken offal or meat	Chicken	1686917	100	48	ST-48 complex
4775	C2257	France		chicken offal or meat	Chicken	1724158	53	572	ST-206 complex
4776	C97anses640	France		carcass swab	Chicken	1607255	30	334	ST-45 complex
4777	CB0004	France		cattle	Ruminants	1611541	67	45	ST-45 complex
4778	CB0011	France		cattle	Ruminants	1658487	36	19	ST-21 complex
4779	CB0012	France		cattle	Ruminants	1664407	41	21	ST-21 complex
4780	CB0013	France		cattle	Ruminants	1648247	39	48	ST-48 complex
4781	CB0023	France		cattle	Ruminants	1696844	46	5359	ST-48 complex
4782	CB0030	France		cattle	Ruminants	1666795	36	21	ST-21 complex
4783	CB0031	France		cattle	Ruminants	1643303	37	586	
4784	CB0061	France		cattle	Ruminants	1647614	40	48	ST-48 complex
4785	CB0072	France		cattle	Ruminants	1657061	33	19	ST-21 complex
4786	CB0084	France		cattle	Ruminants	1650813	68	418	ST-45 complex
4787	CB0089	France		cattle	Ruminants	1771382	90	270	ST-403 complex
4788	CB0091	France		cattle	Ruminants	1636044	42	42	ST-42 complex
4789	CB0096	France		cattle	Ruminants	1665433	86		
4845	ECR105.1	France		caecal sample	Chicken	1695593	98	3574	ST-21 complex
4846	ECR150.1	France		caecal sample	Chicken	1798166	55	6076	ST-45 complex
4887	IFR0079	France		environmental waters	Environment	1710142	345	677	ST-677 complex
4888	IFR0116	France		environmental waters	Environment	1658583	309	2162	
4889	IFR0124	France		environmental waters	Environment	1705663	308	177	ST-177 complex
4890	IFR0152	France		environmental waters	Environment	1612012	124	45	ST-45 complex
4891	IFR0156	France		environmental waters	Environment	1812314	248	5845	
4892	IFR0179	France		environmental waters	Environment	1649458	182	45	ST-45 complex
4893	IFR0186	France		environmental waters	Environment	1850704	65	8571	ST-52 complex
4894	IFR0198	France		environmental waters	Environment	1635844	58	8662	
4895	IFR0234	France		environmental waters	Environment	1691313	268	677	ST-677 complex
4896	IFR0245	France		environmental waters	Environment	1830634	257	5101	
4897	IFR0254	France		environmental waters	Environment	2016840	294	3501	ST-952 complex
4898	IFR0279	France		environmental waters	Environment	1580331	141	6788	ST-1332 complex
4899	IFR0337	France		environmental waters	Environment	1847260	477	1347	ST-1347 complex
4900	IFR0340	France		environmental waters	Environment	1687324	136	1409	
4904	IFR0455	France		environmental waters	Environment	1683993	54	8663	
4905	IFR0457	France		sand (bathing beach)	Environment	1656624	163	2654	
4906	IFR0458	France		sand (bathing beach)	Environment	1631347	54	2654	
4907	IFR0459	France		sand (bathing beach)	Environment	1629447	67	2654	
4908	IFR0585	France		environmental waters	Environment	1846699	279	3218	
4909	IFR0589	France		environmental waters	Environment	1589482	120	3923	
4910	IFR0620	France		environmental waters	Environment	1802740	140	6447	ST-952 complex
4911	IFR0621	France		environmental waters	Environment	1906236	413	6447	ST-952 complex
4913	IFR0800	France		environmental waters	Environment	1780008	275	583	ST-45 complex

Supp. Table 1: Details of the previously published 583 isolates from chicken, ruminant and environment reservoirs used in this study

BIGSdb ID	Strain name	Country of isolation	Year of isolation	Isolation details	Source (for attribution)	genome size	number of contigs	MLST Sequence Type	MLST Clonal Complex
4914	IFR0830	France		environmental waters	Environment	1605469	81	177	ST-177 complex
4916	IFR0839	France		other food	Environment	1730662	298	1959	
4917	IFR0841	France		other food	Environment	1689980	171	1959	
4918	IFR0842	France		other food	Environment	1738522	282	1959	
4919	IFR0870	France		environmental waters	Environment	1600662	49	267	ST-283 complex
4920	IFR0873	France		environmental waters	Environment	1768502	48	2187	ST-508 complex
4921	IFR0949	France		environmental waters	Environment	1645520	71	5707	
4923	IFR1011	France		environmental waters	Environment	1661897	58	45	ST-45 complex
4924	IFR1020	France		environmental waters	Environment	1620416	96	334	ST-45 complex
4925	IFR1108	France		environmental waters	Environment	1767295	136	448	
4926	IFR1152	France		environmental waters	Environment	1591380	52	1286	
4927	IFR1162	France		environmental waters	Environment	1633292	53	2654	
4928	IFR1166	France		environmental waters	Environment	1627686	45	2654	
4929	IFR1223	France		environmental waters	Environment	1713015	77	6076	ST-45 complex
4931	IFR1237	France		environmental waters	Environment	1625274	70	5053	
4932	IFR1268	France		environmental waters	Environment	1551524	43	1316	
6435	C16BOV0002	France	2016	cattle	Ruminants	1708254	358	42	ST-42 complex
6436	C16BOV0060	France	2016	calf	Ruminants	1748912	134	21	ST-21 complex
6437	C16BOV0112	France	2016	cattle	Ruminants	1779371	424	45	ST-45 complex
6438	C16BOV0121	France	2016	cattle	Ruminants	1620375	141	262	ST-21 complex
6439	C16BOV0228	France	2016	calf	Ruminants	1671725	49	3877	ST-21 complex
6440	C16BOV0241	France	2016	calf	Ruminants	1834761	408	990	ST-257 complex
6441	C16BOV0252	France	2016	calf	Ruminants	1767812	98		
6442	C16BOV0279	France	2016	cattle	Ruminants	1643208	33	48	ST-48 complex
6443	C16BOV0336	France	2016	calf	Ruminants	1640787	90		
6444	C16BOV0377	France	2016	cattle	Ruminants	1792877	361	21	ST-21 complex
6445	C16BOV0439	France	2016	cattle	Ruminants	1829627	96	2217	
6446	C16BOV0441	France	2016	cattle	Ruminants	1915288	800	257	ST-257 complex
6447	C16BOV0452	France	2016	calf	Ruminants	1708556	175	122	ST-206 complex
6448	C16BOV0481	France	2016	calf	Ruminants	1838571	429	21	ST-21 complex
6449	C16BOV0553	France	2016	cattle	Ruminants	1827580	436	5707	
6450	C16BOV0606	France	2016	cattle	Ruminants	1785555	469		
6451	C16BOV0611	France	2016	cattle	Ruminants	1788941	430	21	ST-21 complex
6452	C16BOV0674	France	2016	calf	Ruminants	1861059	567	21	ST-21 complex
6453	C16BOV0688	France	2016	calf	Ruminants	1660079	98	19	ST-21 complex
6454	C16BOV0689	France	2016	calf	Ruminants	1796128	491	2230	ST-61 complex
6455	C16BOV0691	France	2016	calf	Ruminants	1857128	409	38	ST-48 complex
6456	C16BOV0705	France	2016	calf	Ruminants	1786845	381	61	ST-61 complex
6457	C16BOV0718	France	2016	cattle	Ruminants	1744662	320	21	ST-21 complex
6458	C16BOV0720	France	2016	cattle	Ruminants	1670220	122	61	ST-61 complex
6459	C16BOV0802	France	2016	calf	Ruminants	1829093	433	21	ST-21 complex
6460	C16BOV0840	France	2016	cattle	Ruminants	1668468	84	21	ST-21 complex
6461	C16BOV0878	France	2016	calf	Ruminants	1695207	65	257	ST-257 complex
6462	C16BOV0973	France	2016	calf	Ruminants	1652552	52	48	ST-48 complex
6463	C16BOV1019	France	2016	cattle	Ruminants	1658844	57	19	ST-21 complex
6464	C16BOV1024	France	2016	cattle	Ruminants	1659248	60		
6465	C16BOV1057	France	2016	cattle	Ruminants	1616542	35	2336	ST-61 complex
6466	C16BOV1058	France	2016	cattle	Ruminants	1737673	80	48	ST-48 complex
6467	C16BOV1071	France	2016	cattle	Ruminants	1689828	94	50	ST-21 complex
6468	C16BOV1073	France	2016	calf	Ruminants	1647003	49	48	ST-48 complex
6469	C16BOV1123	France	2016	cattle	Ruminants	1656524	36	50	ST-21 complex
6470	C16BOV1126	France	2016	calf	Ruminants	1619206	35	658	ST-658 complex
6471	C16BOV1146	France	2016	calf	Ruminants	1789560	291	21	ST-21 complex
6472	C16BOV1168	France	2016	calf	Ruminants	1702013	40	432	ST-61 complex
6473	C16BOV1180	France	2016	cattle	Ruminants	1691042	73	48	ST-48 complex
6474	C16BOV1182	France	2016	cattle	Ruminants	1808741	369	432	ST-61 complex
6475	C16BOV1185	France	2016	cattle	Ruminants	1735919	332	267	ST-283 complex
6476	C16BOV1243	France	2016	cattle	Ruminants	1658120	68	19	ST-21 complex
6477	C16BOV1245	France	2016	cattle	Ruminants	1826143	180	917	ST-21 complex
6478	C16BOV1247	France	2016	cattle	Ruminants	1685870	290	137	ST-45 complex
6479	C16BOV1257	France	2016	calf	Ruminants	1682734	45	122	ST-206 complex
6480	C16BOV1275	France	2016	cattle	Ruminants	1661373	56	61	ST-61 complex
6481	C16BOV1293	France	2016	cattle	Ruminants	1702394	63	21	ST-21 complex
6482	C16BOV1319	France	2016	cattle	Ruminants	1679938	258	61	ST-61 complex
6483	C16BOV1321	France	2016	cattle	Ruminants	1697284	45	21	ST-21 complex
6484	C16BOV1323	France	2016	cattle	Ruminants	1665559	43	38	ST-48 complex
6485	C16BOV1324	France	2016	calf	Ruminants	1725754	214	61	ST-61 complex
6486	C16BOV1337	France	2016	calf	Ruminants	1704594	52	21	ST-21 complex
6487	C16BOV1376	France	2016	cattle	Ruminants	1734272	406	61	ST-61 complex
6488	C16BOV1481	France	2016	cattle	Ruminants	1654324	49	19	ST-21 complex
6489	C16BOV1489	France	2016	cattle	Ruminants	1776595	320	45	ST-45 complex
6490	C16BOV1543	France	2016	calf	Ruminants	1825607	307	42	ST-42 complex
6491	C16BOV1547	France	2016	cattle	Ruminants	1658069	58	61	ST-61 complex
6492	C16BOV1592	France	2016	calf	Ruminants	1755205	68	21	ST-21 complex
6493	C16BOV1600	France	2016	cattle	Ruminants	1651807	38	19	ST-21 complex
6494	C16BOV1602	France	2016	cattle	Ruminants	1668369	77	21	ST-21 complex
6495	C16BOV1668	France	2016	calf	Ruminants	1708115	130	48	ST-48 complex
6496	C16BOV1679	France	2016	calf	Ruminants	1764764	71	19	ST-21 complex
6497	C16BOV1723	France	2016	cattle	Ruminants	1657288	59	21	ST-21 complex
6498	C16BOV1725	France	2016	cattle	Ruminants	1664837	63	21	ST-21 complex
6499	C16BOV1776	France	2016	cattle	Ruminants	1694556	43	257	ST-257 complex
6500	C16BOV1782	France	2016	calf	Ruminants	1744401	95	61	ST-61 complex
6501	C16BOV1813	France	2016	calf	Ruminants	1669936	59	21	ST-21 complex
6502	C16BOV1827	France	2016	cattle	Ruminants	1786955	403	61	ST-61 complex
6503	C16BOV1840	France	2016	cattle	Ruminants	1697409	42	21	ST-21 complex
6504	C16BOV1934	France	2016	calf	Ruminants	1717143	63		
6505	C16BOV1960	France	2016	calf	Ruminants	1831050	53	21	ST-21 complex
6506	C16BOV1977	France	2016	calf	Ruminants	1732281	66	990	ST-257 complex
6507	C16BOV2045	France	2016	calf	Ruminants	1666411	54	61	ST-61 complex
6508	C16BOV2057	France	2016	calf	Ruminants	1760778	49		
6509	C16BOV2151	France	2016	calf	Ruminants	1624222	47	50	ST-21 complex
6510	C16BOV2177	France	2016	calf	Ruminants	1682746	31	61	ST-61 complex
6511	C16BOV2191	France	2016	calf	Ruminants	1663432	42	61	ST-61 complex

Supp. Table 2: Details of the previously published 118 French clinical isolates from 2009 and 2015 used in this stud[illegible]

BIGSdb ID	Strain name	Country of isolation	Year of isolation	Isolation details	genome size	number of contigs	MLST Sequence Type	MLST Clonal Complex
4847	H0026	France	2009	human stool	1712064	78	475	ST-48 complex
4848	H0035	France	2009	human stool	1708151	67	21	ST-21 complex
4849	H0044	France	2009	human stool	1645365	84	475	ST-48 complex
4850	H0058	France	2009	human stool	1708008	44	21	ST-21 complex
4851	H0065	France	2009	human stool	1657192	43	19	ST-21 complex
4852	H0070	France	2009	human stool	1663863	33	46	ST-206 complex
4853	H0079	France	2009	human stool	1706701	38	21	ST-21 complex
4854	H0094	France	2009	human stool	1643034	77	48	ST-48 complex
4855	H0097	France	2009	human stool	1702984	35	5740	ST-206 complex
4856	H0148	France	2009	human stool	1774598	58	2882	ST-353 complex
4857	H0149	France	2009	human stool	1703729	52	21	ST-21 complex
4858	H0198	France	2009	human stool	1755888	78	400	ST-353 complex
4859	H0232	France	2009	human stool	1677559	26	883	ST-21 complex
4860	H0234	France	2009	human stool	1679187	46	1949	ST-21 complex
4861	H0253	France	2009	human stool	1768520	427	21	ST-21 complex
4862	H0271	France	2009	human stool	1636906	52	2262	ST-48 complex
4863	H0296	France	2009	human stool	1711112	79	21	ST-21 complex
4864	H0357	France	2009	human stool	1654469	33	45	ST-45 complex
4865	H0436	France	2009	human stool	1695668	38	45	ST-45 complex
4866	H0450	France	2009	human stool	1655579	46	418	ST-45 complex
4867	H0465	France	2009	human stool	1707647	39	21	ST-21 complex
4868	H0483	France	2009	human stool	1673049	50	290	ST-206 complex
4869	H0544	France	2009	human stool	1752499	70	464	ST-464 complex
4870	H0553	France	2009	human stool	1929060	126	21	ST-21 complex
4871	H0604	France	2009	human stool	1753196	60	464	ST-464 complex
4872	H0624	France	2009	human stool	1717807	66	21	ST-21 complex
4873	H0642	France	2009	human stool	1661406	73	21	ST-21 complex
4874	H0677	France	2009	human stool	1718838	45	572	ST-206 complex
4875	H0679	France	2009	human stool	1708025	75	5751	ST-353 complex
4876	H0701	France	2009	human stool	1628207	22	47	ST-21 complex
4877	H0702	France	2009	human stool	1744098	74	21	ST-21 complex
4878	H0711	France	2009	human stool	1753021	49	464	ST-464 complex
4880	H0783	France	2009	human stool	1692278	64	5	ST-353 complex
4881	H0864	France	2009	human stool	1677872	31	122	ST-206 complex
4882	H0998	France	2009	human stool	1772116	71	21	ST-21 complex
4883	H1003	France	2009	human stool	1659260	56	21	ST-21 complex
4884	H1460	France	2009	human stool	1712075	65	5747	ST-48 complex
4885	H1911	France	2009	human stool	1780009	313	356	ST-353 complex
4886	H2696	France	2009	human stool	1688748	53	353	ST-353 complex
6512	HH0009	France	2015	human stool	1618944	40	905	
6513	HH0106	France	2015	human stool	1714007	38	46	ST-206 complex
6514	HH0117	France	2015	human stool	1748968	89	353	ST-353 complex
6515	HH0122	France	2015	human stool	1660347	58	2254	ST-257 complex
6516	HH0131	France	2015	human stool	1737520	72		
6517	HH0135	France	2015	human stool	1702409	53	21	ST-21 complex
6518	HH0160	France	2015	human stool	1652749	42	22	ST-22 complex
6519	HH0235	France	2015	human stool	1725891	60	2882	ST-353 complex
6520	HH0280	France	2015	human stool	1614639	54	534	
6521	HH0351	France	2015	human stool	1655635	38	2254	ST-257 complex
6522	HH0408	France	2015	human stool	1721201	40	464	ST-464 complex
6523	HH0432	France	2015	human stool	1641366	40	45	ST-45 complex
6524	HH0623	France	2015	human blood culture	1684402	29	299	ST-206 complex
6525	HH0761	France	2015	human stool	1676611	64	3574	ST-21 complex
6526	HH0858	France	2015	human stool	1695471	36	21	ST-21 complex
6527	HH0866	France	2015	human stool	1769746	467	883	ST-21 complex
6528	HH0869	France	2015	human stool	1916399	625	464	ST-464 complex
6529	HH0955	France	2015	human stool	1694094	390		
6530	HH0956	France	2015	human stool	1705222	34	3628	ST-443 complex
6531	HH0990	France	2015	human stool	1601437	59	267	ST-283 complex
6532	HP0003	France	2015	human stool	1684736	63	464	ST-464 complex
6533	HP0023	France	2015	human stool	1685251	25	990	ST-257 complex
6534	HP0119	France	2015	human stool	1747649	47	61	ST-61 complex
6535	HP0200	France	2015	human stool	1620118	31	677	ST-677 complex
6536	HP0219	France	2015	human stool	1674045	48	613	ST-48 complex
6537	HP0231	France	2015	human stool	1755441	312	1519	ST-21 complex
6538	HP0232	France	2015	human stool	1704285	46	917	ST-21 complex
6539	HP0342	France	2015	human stool	1638799	36	52	ST-52 complex
6540	HP0354	France	2015	human stool	1616763	37	50	ST-21 complex
6541	HP0398	France	2015	human stool	1703167	50	19	ST-21 complex
6542	HP0410	France	2015	human stool	1949969	605	354	ST-354 complex
6543	HP0458	France	2015	human stool	1790627	42	2844	ST-460 complex
6544	HP0473	France	2015	human stool	1644144	48	572	ST-206 complex
6545	HP0501	France	2015	human stool	1988298	666	824	ST-257 complex
6546	HP0514	France	2015	human stool	1762539	83	450	ST-446 complex
6547	HP0522	France	2015	human stool	1700769	63	21	ST-21 complex
6548	HP0565	France	2015	human stool	1751212	447	21	ST-21 complex
6549	HP0681	France	2015	human stool	1668916	112	22	ST-22 complex
6550	HP0726	France	2015	human stool	1771385	464	6532	ST-42 complex
6551	HP0727	France	2015	human stool	1818532	34	607	ST-607 complex
6552	HP0766	France	2015	human stool	1674387	37	21	ST-21 complex
6553	HP0826	France	2015	human stool	1650673	63	2100	ST-52 complex
6554	HP0827	France	2015	human stool	1784351	58	523	ST-658 complex
6555	HP0832	France	2015	human stool	1654503	49	22	ST-22 complex
6556	HP0843	France	2015	human stool	1677919	46	1399	
6557	HP0860	France	2015	human stool	1731833	85	464	ST-464 complex
6558	HP0910	France	2015	human stool	1722697	50	148	ST-21 complex
6559	HP0935	France	2015	human stool	1582661	38	42	ST-42 complex
6560	HP0944	France	2015	human stool	1684607	39	5359	ST-48 complex
6561	HP0947	France	2015	human stool	1751488	79	6461	ST-353 complex
6562	HP0973	France	2015	human stool	1728375	45	356	ST-353 complex
6563	HP0988	France	2015	human stool	1597514	42	267	ST-283 complex
6564	HP0990	France	2015	human stool	1601869	43	267	ST-283 complex

Supp. Table 2: Details of the previously published 118 French clinical isolates from 2009 and 2015 used in this study

BIGSdb ID	Strain name	Country of isolation	Year of isolation	Isolation details	genome size	number of contigs	MLST Sequence Type	MLST Clonal Complex
6565	HP1054	France	2015	human stool	1718516	29	122	ST-206 complex
6566	HP1076	France	2015	human stool	1644487	66	25	ST-45 complex
6567	HP1094	France	2015	human stool	1655464	64	49	ST-49 complex
6568	HP1096	France	2015	human stool	1709025	44	44	ST-21 complex
6569	HP1109	France	2015	human stool	1679890	43	48	ST-48 complex
6570	HP1112	France	2015	human stool	1661568	123	48	ST-48 complex
6571	HP1148	France	2015	human stool	1652331	55	45	ST-45 complex
6572	HP1162	France	2015	human stool	1735621	108	353	ST-353 complex
6573	HP1346	France	2015	human stool	1641039	65	6522	ST-443 complex
6574	HP1416	France	2015	human stool	1616217	36	658	ST-658 complex
6575	HP1637	France	2015	human stool	1643723	34	262	ST-21 complex
6576	HP1652	France	2015	human stool	1683663	61	122	ST-206 complex
6577	HP1886	France	2015	human stool	1679644	45	227	ST-206 complex
6578	HP1998	France	2015	human stool	1682045	89	52	ST-52 complex
6579	HP2112	France	2015	human stool	1703971	36	257	ST-257 complex
6580	HP2157	France	2015	human stool	1607249	50		
6581	HP2186	France	2015	human stool	1640357	40	48	ST-48 complex
6582	HP2266	France	2015	human stool	1739520	61	354	ST-354 complex
6583	HP2278	France	2015	human stool	1751596	51	19	ST-21 complex
6584	HP2346	France	2015	human stool	1608757	23	1044	ST-658 complex
6585	HP2353	France	2015	human stool	1657996	46	21	ST-21 complex
6586	HP2440	France	2015	human stool	1892312	639	50	ST-21 complex
6587	HP2456	France	2015	human stool	1772011	393	19	ST-21 complex
6588	HP2555	France	2015	human stool	1856734	448	441	
6589	HP2607	France	2015	human stool	1940487	464	270	ST-403 complex
6590	HP2651	France	2015	human stool	1906579	591	42	ST-42 complex

Supp. Table 3: Details of the 198 French clinical isolates sequenced in this study

BIGSdb ID	Strain name	Year of isolation	Isolation details	Age (year)	Age (month)	Sex	Geographic provenance	Sampling information	genome size	number of contigs	MLST Sequence Type	MLST Clonal Complex
6882	2011_0171	2011	human blood culture	66		female	Vienne(Poitiers)	Private laboratory	1714900	34	3574	ST-21 complex
6883	2011_0198H	2011	human blood culture	5		male	Isère(Voiron)	Public hospital	1697383	14	21	ST-21 complex
6884	2011_0243H	2011	human blood culture	86		female	Vaucluse(Cavaillon)	Public hospital	1691910	14	52	ST-52 complex
6885	2011_0286H	2011	human blood culture	60		male	Vaucluse(Avignon)	Public hospital	1674008	18	122	ST-206 complex
6886	2011_0580	2011	human blood culture	40		female	Seine-et-Marne	Private laboratory	1711719	33	50	ST-21 complex
6887	2011_0584H	2011	human blood culture	65		male	Loire-Atlantique(Nantes)	Public hospital	1699323	42		
6888	2011_0661H	2011	human blood culture	58		male	Gironde(Pessac)	Public hospital	1721717	27	50	ST-21 complex
6889	2011_0951H	2011	human blood culture	3		male	Ardèche(Aubenas)	Public hospital	1834961	34	2274	
6890	2011_1151H	2011	human blood culture	29		female	Gironde(Bordeaux)	Public hospital	1649174	12	48	ST-48 complex
6891	2011_1183H	2011	human blood culture	25		female	Gironde(Bordeaux)	Public hospital	1711000	32	50	ST-21 complex
6892	2011_1200H	2011	human blood culture	49		female	Maine-et-Loire(Saumur)	Public hospital	1674723	21	5173	ST-48 complex
6893	2011_1374H	2011	human blood culture	69		male	Côtes d'Armor(Saint-Brieuc)	Public hospital	1673386	25	21	ST-21 complex
6894	2011_1400H	2011	human blood culture	59		female	Alpes-Maritimes(Nice)	Public hospital	1614157	15	2133	
6895	2011_1433H	2011	human blood culture	60		male	Drôme(Montélimar)	Public hospital	1672850	21	441	
6896	2011_1512H	2011	human blood culture	52		female	Hauts-de-Seine(Garches)	Public hospital	1718420	21	356	ST-353 complex
6897	2011_2753	2011	human blood culture	4		female	Haut-Rhin(Mulhouse)	Private laboratory	1691549	14	61	ST-61 complex
6898	2011_3711	2011	human blood culture	89		male	Indre(Chateauroux)	Private laboratory	1667696	27	2803	
6899	2012_0018H	2012	human blood culture	82		male	Gard(Alès)	Public hospital	1645747	18	48	ST-48 complex
6900	2012_0068H	2012	human blood culture	91		male	Loire-Atlantique(Nantes)	Public hospital	1701730	20	21	ST-21 complex
6901	2012_0090H	2012	human blood culture	23		male	Haute-Vienne(Limoges)	Public hospital	1658219	17	42	ST-42 complex
6902	2012_0215H	2012	human blood culture	52		female	Sarthe(Le Mans)	Public hospital	1581990	15	42	ST-42 complex
6903	2012_0262H	2012	human blood culture	73		female	Haute-Vienne(Limoges)	Public hospital	1667015	24	21	ST-21 complex
6905	2012_0379H	2012	human blood culture	50		male	Vaucluse(Avignon)	Public hospital	1660202	10	8615	ST-61 complex
6906	2012_0667	2012	human blood culture	23		female	Loire-Atlantique(Nantes)	Private laboratory	1641105	21	48	ST-48 complex
6907	2012_0783H	2012	human blood culture	82		male	Lot-et-Garonne	Public hospital	1741152	25	354	ST-354 complex
6908	2012_0995H	2012	human blood culture	56		male	Deux-Sèvres (Thouars)	Public hospital	1730158	45	354	ST-354 complex
6909	2012_1022H	2012	human blood culture	83		female	Seine-Maritime(Elbeuf)	Public hospital	1756532	18	48	ST-48 complex
6910	2012_1030H	2012	human blood culture	91		male	Haute-Vienne(Limoges)	Public hospital	1641117	22	48	ST-48 complex
6911	2012_1069H	2012	human blood culture	67		male	Gironde(Bordeaux)	Public hospital	1798848	48	824	ST-257 complex
6912	2012_1110H	2012	human blood culture	68		male	Gironde(Bordeaux)	Public hospital	1769004	14	2882	ST-353 complex
6914	2012_1131H	2012	human blood culture	71		male	Lot-et-Garonne(Agen)	Public hospital	1688842	21	48	ST-48 complex
6915	2012_1279H	2012	human blood culture	44		female	Gironde(Bordeaux)	Public hospital	1693944	17	257	ST-257 complex
6916	2012_1281H	2012	human blood culture	74		male	Gironde(Bordeaux)	Public hospital	1623050	14	42	ST-42 complex
6917	2012_1416	2012	human blood culture	71		male	Loire-Atlantique(Nantes)	Private laboratory	1644735	16	257	ST-257 complex
6918	2012_3001	2012	human blood culture	1	9	male	Ain	Private laboratory	1666568	12	51	ST-443 complex
6919	2013_0016H	2013	human blood culture	71		female	Manche(Saint lô)	Public hospital	1615543	11	1044	ST-658 complex
6920	2013_0052	2013	human blood culture	35		male	Indre(Chateauroux)	Private laboratory	1651378	12	61	ST-61 complex
6922	2013_0125H	2013	human blood culture	55			Drôme	Public hospital	1743999	15	354	ST-354 complex
6923	2013_0127H	2013	human blood culture	64		male	Marne(Reims)	Public hospital	1676408	24	50	ST-21 complex
6924	2013_0185H	2013	human blood culture	83		female	Haute-Vienne(Limoges)	Public hospital	1683399	24	122	ST-206 complex
6925	2013_0208H	2013	human blood culture	62		male	Haute-Garonne(Toulouse)	Public hospital	1684234	24	48	ST-48 complex
6926	2013_0286H	2013	human blood culture	68		male	Loiret(Orléans)	Public hospital	1636937	28	50	ST-21 complex
6927	2013_0345H	2013	human blood culture	66		female	Haute-Garonne(Toulouse)	Public hospital	1751167	18	572	ST-206 complex
6928	2013_0420H	2013	human blood culture	41		female	Loiret(Orléans)	Public hospital	1691004	19	48	ST-48 complex
6929	2013_0442H	2013	human blood culture	89		male	Loiret(Orléans)	Public hospital	1620669	17	42	ST-42 complex
6930	2013_0449H	2013	human blood culture	10		male	Nord(Lille)	Public hospital	1661977	29	677	ST-677 complex
6931	2013_0465H	2013	human blood culture	88		female	Hérault(Montpellier)	Public hospital	1650423	16	1044	ST-658 complex
6932	2013_0479H	2013	human blood culture	48		male	Haute-Vienne(Limoges)	Public hospital	1613160	16	1044	ST-658 complex
6933	2013_0529H	2013	human blood culture	58		male	Paris(Paris)	Public hospital	1685253	20	299	ST-206 complex
6934	2013_0624H	2013	human blood culture	73		male	Gironde(Pessac)	Public hospital	1654502	23	19	ST-21 complex
6935	2013_0695H	2013	human blood culture	77		male	Haute-Loire(Le puy en velay)	Public hospital	1640827	22	48	ST-48 complex
6936	2013_0724H	2013	human blood culture	50		female	Gironde(Bordeaux)	Public hospital	1761051	27	354	ST-354 complex
6937	2013_0743H	2013	human blood culture	33		female	Manche(Saint lô)	Public hospital	1654717	22	19	ST-21 complex
6938	2013_0775H	2013	human blood culture	69		male	Gard(Alès)	Public hospital	1710486	31	354	ST-354 complex
6939	2013_0886H	2013	human blood culture	57		male	Gironde(Bordeaux)	Public hospital	1781798	24	22	ST-22 complex
6940	2013_0914H	2013	human blood culture	55		female	Moselle(Vantoux)	Public hospital	1739760	27	2066	ST-52 complex
6941	2013_0915H	2013	human blood culture	61		male	Ardèche(Aubenas)	Public hospital	1743365	15	61	ST-61 complex
6942	2013_0936H	2013	human blood culture	81		male	Haute-Loire(Le puy en velay)	Public hospital	1771261	27	48	ST-48 complex
6943	2013_0949H	2013	human blood culture	74		male	Paris(Paris)	Public hospital	1582475	14	42	ST-42 complex
6944	2013_0951H	2013	human blood culture	32		female	Pas-de-Calais(Rang du Fliers)	Public hospital	1660096	26	148	ST-21 complex
6945	2013_1056H	2013	human blood culture	66		female	Loire-Atlantique(Nantes)	Public hospital	1643031	22	572	ST-206 complex
6946	2013_1105H	2013	human blood culture	46		female	Loiret(Orléans)	Public hospital	1672077	20	21	ST-21 complex
6948	2013_1176H	2013	human blood culture	69		male	Gard(Alès)	Public hospital	1748787	38	904	ST-607 complex
6949	2013_1183H	2013	human blood culture	62		male	Drôme(Montélimar)	Public hospital	1785361	21	21,5	ST-21 complex
6950	2013_1249H	2013	human blood culture	73		female	Gironde(Pessac)	Public hospital	1781963	30	904	ST-607 complex
6951	2013_1268	2013	human blood culture	61		male	Loire	Private laboratory	1702617	19	354	ST-354 complex
6952	2013_2151	2013	human blood culture	41		male	Côte-d'Or(Dijon)	Private laboratory	1702834	20	354	ST-354 complex
6953	2013_3388	2013	human blood culture	61		male	Eure-et-Loir(Chartres)	Private laboratory	1782897	27	904	ST-607 complex
6955	2014_0051H	2014	human blood culture	82		male	Pas-de-Calais(Rang du Fliers)	Public hospital	1662077	21	5018	ST-21 complex
6956	2014_0106H	2014	human blood culture	93		female	Gironde(Bordeaux)	Public hospital	1759848	26	2274	
6957	2014_0118H	2014	human blood culture	44		male	Gironde(Langon)	Public hospital	1696539	20		
6958	2014_0241H	2014	human blood culture	59		female	Rhône(Bron)	Public hospital	1645240	15	21	ST-21 complex
6959	2014_0244H	2014	human blood culture	61		male	Hautes-Pyrénées(Tarbes)	Public hospital	1540606	16	5272	
6960	2014_0255H	2014	human blood culture	91		female	Loiret(Orléans)	Public hospital	1683378	13	61	ST-61 complex
6961	2014_0259	2014	human blood culture	74		male	Loire-Atlantique	Private laboratory	1772444	19	122	ST-206 complex
6962	2014_0279H	2014	human blood culture	59		female	Rhône(Bron)	Public hospital	1647358	16		
6963	2014_0289H	2014	human blood culture	76		female	Gironde(Bordeaux)	Public hospital	1637155	27	677	ST-677 complex
6964	2014_0358H	2014	human blood culture	46		male	Lot(Cahors)	Public hospital	1647443	18	52	ST-52 complex
6965	2014_0404H	2014	human blood culture	26		male	Paris(Paris)	Public hospital	1651961	13	2254	ST-257 complex
6966	2014_0422H	2014	human blood culture	63		male	Maine-et-Loire(Cholet)	Public hospital	1642706	19	48	ST-48 complex
6967	2014_0438H	2014	human blood culture	76		male	Alpes-Maritimes	Public hospital	1714642	23	1759	
6968	2014_0450H	2014	human blood culture	54		male	Loiret(Orléans)	Public hospital	1779839	29	464	ST-464 complex
6969	2014_0548H	2014	human blood culture	43		male	Charente-Maritime(La Rochelle)	Public hospital	1640923	17		
6970	2014_0549H	2014	human blood culture	62		male	Gard(Alès)	Public hospital	1626741	28	677	ST-677 complex
6971	2014_0598H	2014	human blood culture	61		female	Loiret(Orléans)	Public hospital	1684731	18	48	ST-48 complex
6972	2014_0748H	2014	human blood culture	49		male	Gironde(Pessac)	Public hospital	1657758	16	81	ST-61 complex
6973	2014_0763H	2014	human blood culture	18		male	Haute-Garonne(Toulouse)	Public hospital	1677430	22	5707	
6974	2014_0886H	2014	human blood culture	69		male	Cantal(Aurillac)	Public hospital	1661998	11	658	ST-658 complex
6975	2014_0889H	2014	human blood culture	31		female	Alpes-Maritimes	Public hospital	1630726	16	883	ST-21 complex
6977	2014_0940	2014	human blood culture	68		male	Indre-et-Loire	Private laboratory	1741699	20	354	ST-354 complex
6978	2014_0950H	2014	human blood culture	83		male	Cantal(Aurillac)	Public hospital	1741361	15	61	ST-61 complex
6979	2014_1005H	2014	human blood culture	73		female	Seine-et-Marne(Meaux)	Public hospital	1617647	14	49	ST-49 complex
6980	2014_1017H	2014	human blood culture	4		male	Loire-Atlantique(Nantes)	Public hospital	1664552	19	21	ST-21 complex
6981	2014_1146H	2014	human blood culture	2	6	female	Alpes-Maritimes	Public hospital	1676907	25	50	ST-21 complex
6982	2014_1209H	2014	human blood culture	23		female	Paris(Paris)	Public hospital	1616989	14	658	ST-658 complex
6983	2014_1220H	2014	human blood culture	88		male	Haute-Vienne(Limoges)	Public hospital	1628590	15	42	ST-42 complex
6984	2014_1299H	2014	human blood culture	66		male	Alpes-Maritimes(Grasse)	Public hospital	1755876	32	354	ST-354 complex
6985	2014_1323	2014	human blood culture	63		male	Cantal(Aurillac)	Private laboratory	1671076	21	1709	ST-1034 complex
6986	2014_2734	2014	human blood culture	36		male	Somme(Doullens)	Private laboratory	1595526	17	267	ST-283 complex
6987	2014_2802	2014	human blood culture	13		male	Haute-Savoie(Cluses)	Private laboratory	1668857	33	5707	
6988	2014_2838	2014	human blood culture	9		male	Loire-Atlantique(Ancenis)	Private laboratory	1706083	24	5718	ST-354 complex
6989	2014_3537	2014	human blood culture	72		female	Haute-Loire(Brioude)	Private laboratory	1672583	23		
6990	2014_3583	2014	human blood culture	10		female	Lot-et-Garonne	Private laboratory	1613708	14	1044	ST-658 complex
6991	2015_0003H	2015	human blood culture	76		male	Maine-et-Loire(Cholet)	Public hospital	1613210	16	1044	ST-658 complex
6992	2015_0048H	2015	human blood culture	77		male	Gironde	Public hospital	1636693	27	677	ST-677 complex
6993	2015_0050H	2015	human blood culture	78		male	Moselle(Metz)	Public hospital	1733804	48	44	ST-21 complex
6994	2015_0061H	2015	human blood culture	48		male	Paris(Paris)	Public hospital	1657565	13	61	ST-61 complex
6995	2015_0063H	2015	human blood culture	49		male	Gironde	Public hospital	1615330	14	1044	ST-658 complex
6996	2015_0068H	2015	human blood culture	80		male	Loiret(Orléans)	Public hospital	1677139	25	50	ST-21 complex
6997	2015_0109H	2015	human blood culture	85		female	Seine-Maritime(Elbeuf)	Public hospital	1747880	18	354	ST-354 complex
6998	2015_0204H	2015	human blood culture	56		female	Paris(Paris)	Public hospital	1691345	19	257	ST-257 complex
7000	2015_0334H	2015	human blood culture	73		male	Vendée(La roche sur Yon)	Public hospital	1595220	19	538	ST-45 complex
7001	2015_0345H	2015	human blood culture	8		male	Loiret(Orléans)	Public hospital	1713903	29	2641	ST-353 complex
7002	2015_0374H	2015	human blood culture	80		male	Hautes-Pyrénées(Tarbes)	Public hospital	1692877	8	990	ST-257 complex
7003	2015_0450H	2015	human blood culture	64		male	Vaucluse(Avignon)	Public hospital	1637303	15	1721	
7004	2015_0505H	2015	human blood culture	78		male	Ardèche(Aubenas)	Public hospital	1706124	25	6461	ST-353 complex
7005	2015_0518H	2015	human blood culture	14		male	Loire-Atlantique(Nantes)	Public hospital	1737484	28	353	ST-353 complex
7006	2015_0612	2015	human blood culture	33		female	Haut-Rhin(Mulhouse)	Private laboratory	1635627	18	6522	ST-443 complex
7007	2015_0685H	2015	human blood culture	50		male	Ardèche(Annonay)	Public hospital	1657254	16	49	ST-49 complex
7008	2015_0737H	2015	human blood culture	11		male	Gironde(Langon)	Public hospital	1726655	30	464	ST-464 complex
7010	2015_0757H	2015	human blood culture	70		male	Ain(Bourg en Bresse)	Public hospital	1683631	24	50	ST-21 complex
7011	2015_0769H	2015	human blood culture	67		male	Maine-et-Loire(Cholet)	Public hospital	1651117	15	1044	ST-658 complex
7012	2015_0795H	2015	human blood culture	72		female	Vaucluse(Avignon)	Public hospital	1682858	30	50	ST-21 complex
7013	2015_0808H	2015	human blood culture	58		male	Vendée(La roche sur Yon)	Public hospital	1622115	15	2109	ST-45 complex
7014	2015_0824H	2015	human blood culture	59		male	Haute-Loire(Le puy en velay)	Public hospital	1574183	17		
7015	2015_0859H	2015	human blood culture	46		female	Moselle(Metz)	Public hospital	1618950	15	658	ST-658 complex
7016	2015_0959H	2015	human blood culture	25		female	Haute-Vienne(Limoges)	Public hospital	1657730	20	50	ST-21 complex
7017	2015_0981H	2015	human blood culture	0	4	male	Loire-Atlantique(Nantes)	Public hospital	1646233	10	48	ST-48 complex

Supp. Table 3: Details of the 198 French clinical isolates sequenced in this study

BIGSdb ID	Strain name	Year of isolation	Isolation details	Age (year)	Age (month)	Sex	Geographic provenance	Sampling information	genome size	number of contigs	MLST Sequence Type	MLST Clonal Complex
7018	2015_1053H	2015	human blood culture	76		male	Vendée(La roche sur Yon)	Public hospital	1662309	23	441	
7019	2015_1056H	2015	human blood culture	20		female	Nord(Tourcoing)	Public hospital	1722892	21	353	ST-353 complex
7020	2015_1064H	2015	human blood culture	79		female	Gironde	Public hospital	1657305	17	45	ST-45 complex
7021	2015_1368	2015	human blood culture	19		female	Saône-et-Loire	Private laboratory	1677765	21	572	ST-206 complex
7022	2015_1581	2015	human blood culture	81		male	Haute-Garonne(Montrejeau)	Private laboratory	1683314	10		
7023	2015_1831	2015	human blood culture	55		male	Loire-Atlantique(Trignac)	Private laboratory	1613135	14	1044	ST-658 complex
7024	2015_1979	2015	human blood culture	79		male	Loire-Atlantique(Trignac)	Private laboratory	1681238	18	257	ST-257 complex
7025	2016_0006H	2016	human blood culture	48		male	Finistère	Public hospital	1654452	12	257	ST-257 complex
7026	2016_0018H	2016	human blood culture	69		female	Vendée(La roche sur Yon)	Public hospital	1707398	17	110	ST-21 complex
7027	2016_0036H	2016	human blood culture	60		female	Haute-Vienne(Limoges)	Public hospital	1611208	14	61	ST-61 complex
7028	2016_0062H	2016	human blood culture	48		male	Lot-et-Garonne	Public hospital	1654503	13	257	ST-257 complex
7029	2016_0148	2016	human blood culture	1	1	female	Paris(Paris)	Private laboratory	1659642	17	21	ST-21 complex
7030	2016_0183H	2016	human blood culture	39		male	Ardennes(Charleville Mézières)	Public hospital	1616656	25	22	ST-22 complex
7031	2016_0232H	2016	human blood culture	89		male	Loire-Atlantique(Nantes)	Public hospital	1706428	16	3155	ST-354 complex
7033	2016_0335H	2016	human blood culture	54		male	Moselle(Metz)	Public hospital	1757208	35	464	ST-464 complex
7034	2016_0488H	2016	human blood culture	53		male	Loiret(Orléans)	Public hospital	1727063	28	464	ST-464 complex
7035	2016_0589H	2016	human blood culture	83		female	Drôme(Montélimar)	Public hospital	1672791	13	52	ST-52 complex
7036	2016_0605H	2016	human blood culture	0	0	female	Haute-Vienne(Limoges)	Public hospital	1730561	31	353	ST-353 complex
7037	2016_0609	2016	human blood culture	65		male	Vaucluse(Roussillon)	Private laboratory	1624528	19	7436	ST-21 complex
7038	2016_0619H	2016	human blood culture	55		male	Ardèche(Privas)	Public hospital	1717713	20	1301	ST-692 complex
7039	2016_0638H	2016	human blood culture	85		male	Rhône(Lyon)	Public hospital	1793086	48	2274	
7040	2016_0750H	2016	human blood culture	29		female	Essonne(Corbeil-Essonnes)	Public hospital	1660483	22	19	ST-21 complex
7041	2016_0751H	2016	human blood culture	4		male	Loire-Atlantique(Nantes)	Public hospital	1669953	24		
7042	2016_0753H	2016	human blood culture	92		male	Isère(Bourgoin Jallieu)	Public hospital	1668275	18	52	ST-52 complex
7043	2016_0771H	2016	human blood culture	59		female	Rhône(Bron)	Public hospital	1619278	12	49	ST-49 complex
7044	2016_0777H	2016	human blood culture	87		female	Vendée(La roche sur Yon)	Public hospital	1688777	16	257	ST-257 complex
7045	2016_0810H	2016	human blood culture	70		male	Vendée(La roche sur Yon)	Public hospital	1628793	18	21	ST-21 complex
7046	2016_0817H	2016	human blood culture	16		male	Côtes d'Armor(Saint Brieuc)	Public hospital	1726109	10	48	ST-48 complex
7047	2016_0857	2016	human blood culture	49		male	Pas-de-Calais(Henin Beaumont)	Private laboratory	1679620	14	436	
7048	2016_0873H	2016	human blood culture	29		male	Hérault(Montpellier)	Public hospital	1633970	15	51	ST-443 complex
7049	2016_0891H	2016	human blood culture	87		male	Drôme(Montélimar)	Public hospital	1634770	14	6522	ST-443 complex
7050	2016_0900H	2016	human blood culture	60		female	Essonne(Corbeil-Essonnes)	Public hospital	1635529	20	6522	ST-443 complex
7051	2016_0998	2016	human blood culture	35		male	Vendée(Mortagne-Sur-Sevre)	Private laboratory	1722782	31	464	ST-464 complex
7052	2016_1009	2016	human blood culture	87		male	Gironde(Langon)	Private laboratory	1677825	18	61	ST-61 complex
7053	2016_1064H	2016	human blood culture	76		female	Lot(Cahors)	Public hospital	1602123	12		
7054	2016_1070H	2016	human blood culture	2	11	male	Gard(Bagnols sur Ceze)	Public hospital	1672774	15	51	ST-443 complex
7055	2016_1437	2016	human blood culture	65		male	Doubs(Besancon)	Private laboratory	1654389	22	19	ST-21 complex
7056	2016_1464	2016	human blood culture	12		female	Corrèze(Brive La Gaillarde)	Private laboratory	1656849	20	19	ST-21 complex
7057	2016_1465	2016	human blood culture	2	3	female	Haute-Garonne(Montrejeau)	Private laboratory	1642431	17	52	ST-52 complex
7058	2016_2202	2016	human blood culture	82		male	Vaucluse(Carpentras)	Private laboratory	1640472	20	122	ST-206 complex
7059	2016_2420	2016	human blood culture	20		female	Nord(Lille)	Private laboratory	1613635	13	775	ST-52 complex
7060	2016_2698	2016	human blood culture	81		male	Saône-et-Loire	Private laboratory	1737381	16	1900	ST-658 complex
7061	2016_3084	2016	human blood culture	20		male	Morbihan(Vannes)	Private laboratory	1662957	28	49	ST-49 complex
7062	2016_3144	2016	human blood culture	63		male	Saône-et-Loire	Private laboratory	1789051	26	7959	
7063	2016_3412	2016	human stool	9		male	Seine-Maritime(Barentin)	Private laboratory	1788692	34	607	ST-607 complex
7064	2016_3415	2016	human stool	27		male	Maine-et-Loire	Private laboratory	1690212	19	257	ST-257 complex
7066	2016_3419	2016	human stool	6		male	Ain(Bourg-En-Bresse)	Private laboratory	1682359	31	5018	ST-21 complex
7067	2016_3420	2016	human stool	65		male	Ardennes(Rethel)	Private laboratory	1646629	17	1519	ST-21 complex
7068	2016_3421	2016	human stool	22		male	Aude(Narbonne)	Private laboratory	1655982	15	21	ST-21 complex
7069	2016_3422	2016	human stool	19		male	Haute-Saône(Hericourt)	Private laboratory	1653779	12	2254	ST-257 complex
7070	2016_3423	2016	human stool	46		female	Haut-Rhin(Wittenheim)	Private laboratory	1709333	23	50	ST-21 complex
7071	2016_3427	2016	human stool	35		male	Oise(Liancourt)	Private laboratory	1654309	19	19	ST-21 complex
7072	2016_3429	2016	human stool	18		male	Maine-et-Loire	Private laboratory	1665737	13	6522	ST-443 complex
7073	2016_3430	2016	human stool	23		female	Paris(Paris)	Private laboratory	1709955	18	354	ST-354 complex
7074	2016_3431	2016	human stool	13		male	Essonne(Longjumeau)	Private laboratory	1686182	16	52	ST-52 complex
7075	2016_3432	2016	human stool	6		female	Haute-Garonne(Montrejeau)	Private laboratory	1588742	17	42	ST-42 complex
7076	2016_3433	2016	human stool	60		male	Nord(Hazebrouck)	Private laboratory	1644072	36	2861	
7077	2016_3434	2016	human stool	9		female	Haute-Saône(Hericourt)	Private laboratory	1725115	30	464	ST-464 complex
7078	2016_3437	2016	human stool	54		female	Morbihan(Vannes)	Private laboratory	1702486	18	21	ST-21 complex
7079	2016_3438	2016	human stool	89		male	Morbihan(Vannes)	Private laboratory	1621092	15	42	ST-42 complex
7080	2016_3439	2016	human stool	47		male	Corrèze(Uzerche)	Private laboratory	1705386	18	354	ST-354 complex
7081	2016_3440	2016	human stool	7		female	Val-de-Marne(Saint Mande)	Private laboratory	1652997	21	19	ST-21 complex
7082	2016_3441	2016	human stool	1	0	female	Loire-Atlantique(Trignac)	Private laboratory	1611528	14	137	ST-45 complex
7083	2016_3442	2016	human stool	77		male	Saône-et-Loire	Private laboratory	1616611	24	22	ST-22 complex
7084	2016_3443	2016	human stool	37		male	Aveyron(Rodez)	Private laboratory	1616404	17	49	ST-49 complex
7085	2016_3446	2016	human stool	31		female	Corrèze(Brive la gaillarde)	Private laboratory	1642981	15	52	ST-52 complex
7086	2016_3449	2016	human stool	14		male	Ardennes(Rethel)	Private laboratory	1616654	15	2314	ST-1034 complex
7087	2016_3451	2016	human stool	1	1	female	Loire(Saint Chamond)	Private laboratory	1630958	13	469	ST-42 complex
7088	2016_3452	2016	human stool	29		male	Loire(Saint Chamond)	Private laboratory	1694867	26	6175	ST-21 complex
7089	2016_3453	2016	human stool	27		female	Haut-Rhin(Wittenheim)	Private laboratory	1639601	10	3172	ST-574 complex

Supp. Table 4: List of the 15 host-segregating markers used for attribution in this study

Locus tag	Alias	Gene	Genome	Size of the gene
Cj0133	CAMP0121		Core	402
Cj0260c	CAMP0230		Accessory	216
Cj0348	CAMP0314	trpB	Core	1179
Cj0350	CAMP0316		Soft Core	423
Cj0663c	CAMP0615	hslV	Core	543
Cj0986c	CAMP0909		Soft Core	189
Cj1047c	CAMP0970	thiS	Core	192
Cj1169c	CAMP1088		Core	228
Cj1186c	CAMP1105	petA	Soft Core	504
Cj1192	CAMP1111	dctA	Soft Core	1377
Cj1204c	CAMP1123	atpB	Soft Core	681
Cj1220	CAMP1139	groES	Soft Core	261
Cj1463	CAMP1373	flgJ	Core	342
Cj1513c	CAMP1419		Core	192
Cj1621	CAMP1517		Soft Core	753
